# Insights on Zosteric Acid Analogues Activity Against Candida albicans Biofilm Formation

**DOI:** 10.1021/acsomega.5c03581

**Published:** 2025-05-20

**Authors:** Cristina Cattò, Enrico M. A. Fassi, Giovanni Grazioso, Arianna Gelain, Stefania Villa, Francesca Cappitelli

**Affiliations:** † Department of Food, Environmental and Nutritional Sciences, 9304 University of Milan, via G. Celoria 2, 20133 Milan, Italy; ‡ Department of Pharmaceutical Sciences, University of Milan, via L. Mangiagalli 25, 20133 Milan, Italy

## Abstract

Zosteric acid (ZA), or *p*-(sulphooxy)­cinnamic acid, is a secondary metabolite of the seagrass Zostera marina able to reduce biofilm formation of a wide range of bacteria and fungi, through a nonbiocidal mode of action. However, the lack of information concerning the specific chemical structural elements responsible for ZA’s antibiofilm activity has hindered the scaling up of this green-based technology for real applications. In this study, a small library of molecules based on ZA scaffold diversity was screened against the eukaryotic fungus Candida albicans, in order to identify the key chemical features of ZA necessary for inhibiting fungal biofilm at sublethal concentrations. Results, supported by multivariate statistical analysis, revealed that the presence of (i) the *trans* (*E*) double bond, (ii) the free carboxylic group in the side chain, and (iii) the *para* substitution with a hydroxyl group were all instrumental for maintaining the antibiofilm activity of the molecules. Additionally, molecular modeling studies suggested that the best performing derivatives interacted with NADP­(H) quinone oxidoreductase, influencing the microbial redox balance.

## Graphical abstract

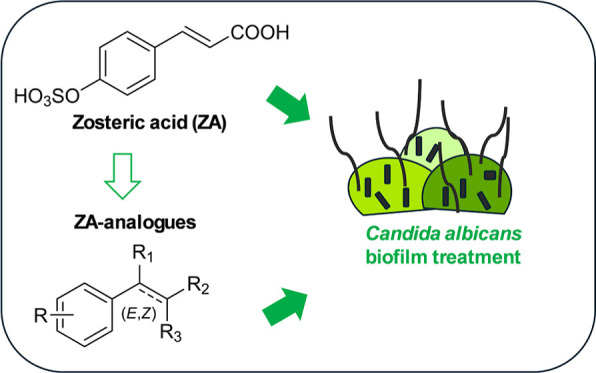

## Introduction

It is widely recognized that most fungi grow in the form of biofilms, which are complex structured sessile communities of microorganisms embedded in a self-produced polymeric matrix. The impact of fungal biofilms on present-day life is incalculable as their removal from surfaces is difficult or even impossible. In their sessile lifestyle, fungi have significantly increased tolerance to antimicrobial and cleaning agents, and this constitutes a great concern throughout the different spheres of the economy, such as agri-food, industrial and medical sectors.^1^ Additionally, regulations are becoming more restrictive, leading to the banning of several biocides hazardous to human health and the environment.^2^

In the past decade, alternative sustainable approaches perceived by the public as safe have been proposed. These approaches involve natural molecules that are capable of depriving microorganisms of their ability to develop a biofilm without killing them. They act at sublethal doses, reducing the selection pressure for drug-resistant mutations.^3^ Despite a significant amount of work being devoted to the discovery of these biofilm inhibitors, relatively little research has focused on understanding the structural elements required for their antibiofilm activity and their mechanism of action at a cellular level. This information is crucial for fully exploiting these molecules in real-world settings.^4^

Zosteric acid (ZA), or *p*-(sulphooxy)­cinnamic acid, is a secondary metabolite of seagrass Zostera marina. ZA has been extensively studied for its nonbiocidal antibiofilm activity against a number of fungi, including Colletotrichum lindemuthianum, Magnaporthe grisea, Aspergillus niger, Penicillium citrinum, and Candida albicans.^5–8^ Villa et al.^7^ showed that 10 mg/L ZA significantly impacted the thickness and morphology of C. albicans fungal biofilms, leading cells to the inability to form filamentous structures. Recently, Cattò et al.^9^ scanned the entire C. albicans proteome and identified, for the first time, the fungal proteins involved in ZA activity. Upon ZA treatment, proteins involved in structure, integrity, and biogenesis of cell walls as well as those involved in adhesion and stable attachment of hyphae were found to be downregulated, while some proteins involved in the stress response were found to be overexpressed.

The application of ZA in a real setting is very promising. ZA displays low toxicity toward organisms, human cells, and the environment.^7,10^ The simple chemical structure allows its chemical synthesis with a satisfying purification level, avoiding the need to harvest marine plants and relying on seasonally dependent availability. Recently, a biological process based on the genetic engineering of microorganisms has been proposed as an alternative and preferred system for the production of this plant chemical, with a system that eliminates harsh chemical conditions and reduces waste generation.^11^

However, the green ZA-based technology against fungal biofilms is far from a real application. The lack of knowledge regarding the exact chemical structural elements responsible for ZA’s antibiofilm activity is one of the limiting factors in the development of ZA-technologies. Structural information allows new molecule-based applications and material improvements. For example, the understanding of ZA’s structural functional group could be exploited to link ZA to an abiotic surface, delivering its active moiety to the fungal cellular target. Moreover, the knowledge of the ZA active moiety could be used to generate more potent derivatives based on the same structure. A study by Cattò et al.^12^ concluded that the cinnamic acid scaffold is responsible for ZA’s antibiofilm performance against the bacterium Escherichia coli.

In this study, a small library of molecules based on the ZA scaffold diversity was screened against the eukaryotic C. albicans in order to understand the ZA chemical structural determinants necessary for inhibiting fungal biofilm at sublethal concentrations. C. albicans was chosen as a cellular model due to its unique features in terms of life cycle, genome structure, and dynamics, including the ability to colonize different settings, from the food industry to hospitals.^13^ Finally, to provide an explanation of the probable action mechanism, the most active compounds were simulated in complex with a NADP­(H) quinone oxidoreductase virtual model, since ZA seems to be involved in the cellular redox balance.^9^

## Results and Discussion

Structural determinants of ZA necessary for its activity against fungal biofilms have been investigated in order to highlight features that could be exploited to scale up ZA in real settings as well as to design new analogues with improved activity. Following this perspective, a small library of compounds (Figure 1, Table 1) was designed, starting from the chemical structure of ZA and introducing modifications by (i) changing the type and position of substituents on the phenyl ring; (ii) replacing the carboxylic acid function of the side chain with aldehyde, ester, and alcohol functionalities or completely removing it; (iii) considering both *E*/*Z* isomers and the corresponding saturated derivatives to study the role of the double bond (Figure 1). The library consisted of synthesized (**1**, **2**, **4**, **6**, **27**, and **28**) and commercially available (**3**, **5**, **7**–**26**, **29**–**31**) compounds.

**Figure 1:**
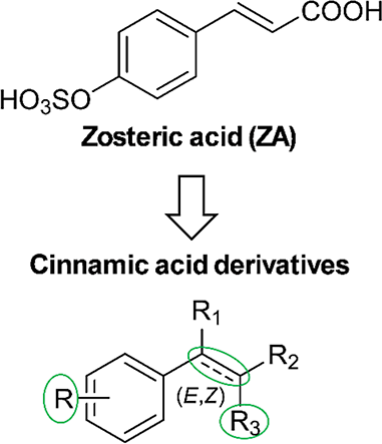
Modifications introduced on ZA structure.

**Table 1:**
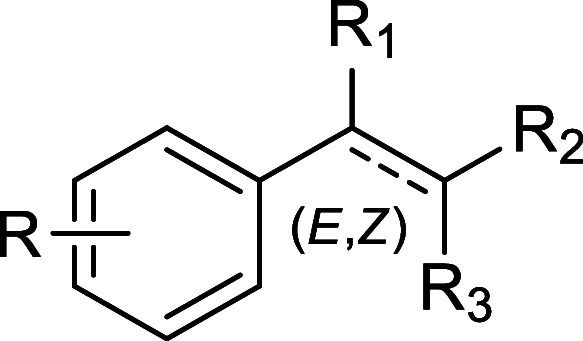
ZA-Analogues Tested against C. albicans Biofilm Formation

compound	**R**	**R**_**1**_	**R**_**2**_	**R**_**3**_	configuration
1 (ZA)	4-OSO_3_H	H	H	COOH	*E*
2	4-OSO_3_H	H	H	COOH	*Z*
3	H	H	H	COOH	*E*
4	H	H	H	COOH	*Z*
5	H	H,H	H,H	COOH	-
6	H	H	H	COOCH_3_	*E*
7	H	H	H	COOCH_2_CH_3_	*E*
8	H	CH_3_	H	COOCH_2_CH_3_	*E*
9	2-OH	H	H	COOCH_2_CH_3_	*E*
10	H	H	H	COH	*E*
11	H	H	CH_3_	COH	*E*
12	H	H	H	CH_2_OH	*E*
13	4-OH	H	H	COOH	*E*
14	4-OH	H	H	COOH	*Z*
15	4-OH	H,H	H,H	COOH	-
16	4-OH	H	H	COOCH_3_	*E*
17	3-OH	H	H	COOH	*E*
18	2-OH	H	H	COOH	*E*
19	3,4-OH	H	H	COOH	*E*
20	4-OCH_3_	H	H	COOH	*E*
21	2-OCH_3_	H	H	COOH	*E*
22	4-Cl	H	H	COOH	*E*
23	3-Cl	H	H	COOH	*E*
24	3,4-Cl	H	H	COOH	*E*
25	2,4-Cl	H	H	COOH	*E*
26	2-Cl,4-F	H	H	COOH	*E*
27	4-CH_3_	H	H	COOH	*E*
28	4-COOH	H	H	COOH	*E*
29	4-CHO	H	H	COOH	*E*
30	4-NO_2_	H	H	COOH	*E*
31	4-NH_2_.HCl	H	H	COOH	*E*

Biological activities of ZA analogues have been compared with data obtained with ZA, using 35 μM as the reference concentration because a previous study demonstrated that 35 μM was the best concentration of ZA able to affect C. albicans biofilm formation.^7,9^

### Carbon and Energy Source

Before evaluating the antibiofilm properties of ZA-analogues, the ability of C. albicans to use them as a carbon and energy source was tested in order to exclude that any adhesion assay results might be attributed to a nutrient function instead of a biological activity, as otherwise the molecule increases biofilm development instead of preventing it.^14^ The experiment showed that C. albicans was unable to grow in the mineral medium supplemented with each ZA-derivative. In contrast, the yeast grew in the mineral medium supplemented with 10 mM glucose (Table S1). Therefore, any pro-biofilm activity of ZA-analogues should be attributed to mechanisms other than their function as growth substrates.

### Planktonic Growth

Finding molecules that exploit antibiofilm activity without impacting cell viability, but instead downregulating the expression of virulence genes involved in biofilm formation, is a current milestone in science.^15^ Indeed, microorganisms develop resistance to any drugs affecting their viability and this is a major problem worldwide.^16^ By acting at sublethal concentrations, molecules exert their activity reducing the selection pressure that leads to resistant strains.^14^ Therefore, the ability of each ZA-derivative to affect the viability of C. albicans was examined to confirm the absence of killing activity.

The inhibitory effect of each compound on fungal growth was studied by using a 24 h planktonic kinetic profile. This time-resolved growth analysis is more realistic experimentally than the conventional toxicological assessments that focus on a single end point determination of optical density after 24 h of growth.^17^ Absorbance-based growth kinetics were constructed for each molecule, and the resulting curves are reported in Figure 2.

**Figure 2:**
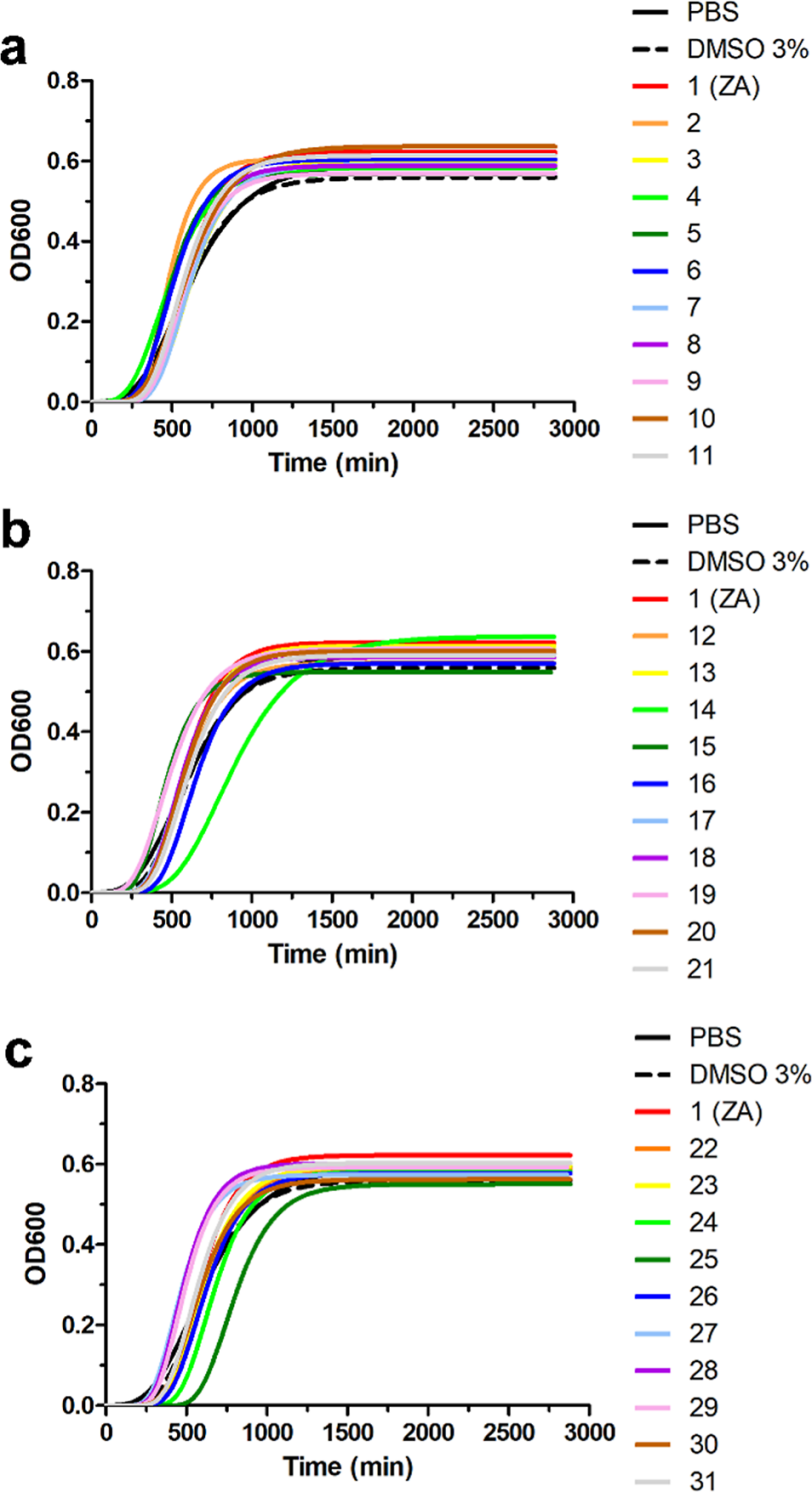
C. albicans planktonic growth without and with ZA-analogues. Graphs represent the average growth curves of C. albicans obtained from OD600 data over time and fitted with the Gompertz model. Panel A shows compounds **1**–**11**, panel B shows compounds **12**–**21**, and panel C shows compounds **22**–**31**. In each panel, positive (1) and negative (PBS, and DMSO 3%) controls are also reported.

The Zwietering-modified version of the Gompertz polynomial model was used to analyze the toxicological effects of each compound on the kinetic parameters. The exponential maximum growth rate (μ_m_) and the end point of kinetic growth (Y_M_), which corresponds to the maximum fungal growth during the stationary phases, were considered direct measures of vitality.^18^ It has been reported that changes in environmental conditions can trigger a shift in microbial communities by causing a decrease in μ_m_ when microbial cells face adverse conditions.^19^ Statistical analysis performed with μ_m_ values showed that compounds **14** and **25** decreased the growth rate of C. albicans during the exponential phase (Table 2). Specifically, the presence of compound **14** led to a significant reduction in fungal growth, with a decrease in growth rate of −40.5% compared to the negative control with 3% DMSO. Compound **25** also decreased fungal growth by −11.8% compared with the same control. Furthermore, ANOVA analysis (Table 2) did not reveal any statistical differences in the Y_M_ values of all ZA-analogues compared to the negative control with 3% DMSO.

**Table 2:** C. albicans Planktonic Growth without (PBS and DMSO 3%) and with ZA-Analogues^a^

compound	λ [min]	μ_m_ [OD600/min]	Y_M_ [OD600]
PBS	255.9 ± 9.3 [−5.4]	(1.57 ± 0.13) × 10^–3^ [2.9]	0.536 ± 0.024 [−8.7]
DMSO 3%	270.6 ± 13.9 [0.0]	(1.53 ± 0.03) × 10^–3^ [0.0]	0.587 ± 0.016 [0.0]
1 (ZA)	269.3 ± 35.0 [−0.5]	(1.66 ± 0.05) × 10^–3^ [8.8]	0.634 ± 0.036 [8.1]
2	302.1 ± 11.0 [11.7]*	(1.64 ± 0.07) × 10^–3^ [7.6]	0.637 ± 0.049 [8.5]
3	251.6 ± 2.4 [−7.0]	(1.50 ± 0.05) × 10^–3^ [−1.6]	0.606 ± 0.003 [3.3]
4	296.1 ± 10.9 [9.5]	(1.62 ± 0.13) × 10^–3^ [6.4]	0.589 ± 0.035 [0.3]
5	286.6 ± 3.2 [5.9]	(1.45 ± 0.07) × 10^–3^ [−4.9]	0.547 ± 0.034 [−6.7]
6	208.6 ± 2.2[−22.9]*	(1.61 ± 0.05) × 10^–3^ [5.5]	0.613 ± 0.013 [4.4]
7	252.0 ± 4.4 [−6.9]	(1.52 ± 0.05) × 10^–3^ [−0.4]	0.602 ± 0.017 [2.6]
8	241.4 ± 1.2 [−10.8]*	(1.64 ± 0.03) × 10^–3^ [7.5]	0.606 ± 0.004[3.2]
9	242.5 ± 5.7 [−10.4]*	(1.55 ± 0.03) × 10^–3^ [1.7]	0.587 ± 0.013 [0.0]
10	251.4 ± 0.5[−7.1]	(1.43 ± 0.04) × 10^–3^ [−6.3]	0.636 ± 0.056 [8.4]
11	243.6 ± 12.1 [−10.0]	(1.48 ± 0.03) × 10^–3^ [−2.8]	0.631 ± 0.104 [7.5]
12	242.9 ± 4.6 [−10.2]	(1.50 ± 0.04) × 10^–3^ [−1.9]	0.583 ± 0.018[−0.7]
13	240.0 ± 2.2 [−11.3]	(1.57 ± 0.06) × 10^–3^ [3.0]	0.629 ± 0.018 [7.1]
14	303.4 ± 2.6 [12.1]*	(0.91 ± 0.03) × 10^–3^ [−40.5]*	0.644 ± 0.048 [9.8]
15	282.4 ± 4.1 [4.4]	(1.51 ± 0.03) × 10^–3^ [−1.1]	0.549 ± 0.006 [−6.4]
16	269.3 ± 12.0 [−0.5]	(1.44 ± 0.04) × 10^–3^ [−5.5]	0.580 ± 0.023 [−1.2]
17	233.4 ± 2.1 [−13.7]*	(1.54 ± 0.05) × 10^–3^ [0.8]	0.618 ± 0.007 [5.4]
18	238.4 ± 2.9 [−11.9]	(1.61 ± 0.01) × 10^–3^ [5.9]	0.603 ± 0.008 [2.8]
19	285.9 ± 14.0 [5.7]	(1.50 ± 0.07) × 10^–3^ [−1.5]	0.577 ± 0.023 [−1.7]
20	240.5 ± 2.9 [−11.1]*	(1.58 ± 0.01) × 10^–3^ [3.6]	0.618 ± 0.012 [5.3]
21	246.5 ± 3.8 [−8.9]	(1.49 ± 0.05) × 10^–3^ [−2.6]	0.602 ± 0.014 [2.6]
22	245.0 ± 1.4 [−9.5]	(1.48 ± 0.02) × 10^–3^ [−3.3]	0.599 ± 0.004 [2.2]
23	241.7 ± 5.7 [−10.7]*	(1.47 ± 0.02) × 10^–3^ [−3.3]	0.603 ± 0.013 [2.8]
24	284.4 ± 3.5 [5.1]	(1.52 ± 0.04) × 10^–3^ [0.0]	0.594 ± 0.017 [1.2]
25	337.4 ± 1.2 [24.7]*	(1.34 ± 0.04) × 10^–3^ [−11.8]*	0.565 ± 0.008 [−3.6]
26	255.0 ± 2.4 [−5.7]	(1.45 ± 0.01) × 10^–3^ [−4.6]	0.596 ± 0.006 [1.5]
27	285.3 ± 4.8 [5.5]	(1.58 ± 0.05) × 10^–3^ [3.5]	0.573 ± 0.017 [−2.3]
28	300.6 ± 10.9 [11.1]*	(1.60 ± 0.02) × 10^–3^ [5.0]	0.607 ± 0.020 [3.5]
29	289.2 ± 10.2 [6.9]	(1.43 ± 0.05) × 10^–3^ [−6.2]	0.585 ± 0.016 [−0.2]
30	235.9 ± 0.8 [−12.8]*	(1.42 ± 0.01) × 10^–3^ [−6.6]	0.579 ± 0.015 [−1.2]
31	240.0 ± 1.9 [−11.3]	(1.64 ± 0.02) × 10^–3^ [7.2]	0.621 ± 0.011 [5.9]

a Data report growth parameters obtained by the Gompertz model: lag time length (λ), exponential maximum growth rate (μ_m_), and the end point of kinetic growth (Y_M_). Data represent the mean ± standard deviation of at least three independent measurements. Values in squared brackets indicate the percentage increase/decrease compared to the negative control obtained with 3% DMSO. A star indicates statistically significant differences (Tukey’s honestly significant difference (HSD), *p* ≤ 0.05) between the value and the negative control with 3% DMSO.

Recent studies suggested that the duration of the lag phase (λ) is equally important in assessing the fitness and stress tolerance of microbial populations.^20^ Therefore, in this study, the length of λ was considered as an indicator of microbial viability along with μ_m_ and Y_M_, and it was calculated for each ZA-derivative (Table 2).

ANOVA analysis revealed that compounds **14**, **25**, and **28** statistically increased the length of the lag phase (Table 2). Compound **25** showed the most consistent increase, with a +24.7% compared to the negative control with 3% DMSO, while compounds **14** and **28** were 12.1% and 11.2% higher, respectively, compared to the negative control with 3% DMSO. It is believed that the lag phase allows bacterial cells to adapt to new environmental conditions. Although lag is a temporary period of nonreplication, microbial cells are active, and their metabolism includes the activation of pathways and processes necessary for subsequent cell division. When cells face environmental challenges and stress levels rise, they may require longer periods of adjustment, leading to a prolonged lag phase, as specific genes or enzymes are expressed to keep cellular systems functional before cell division begins.^20^ Indeed, research has shown that the extension of the lag time is an ecological adaptive trait that provides protection in response to stress exposure.^21^

On the contrary, molecules **6**, **8**, **9**, **12**, **17**, **20**, **23**, and **30** decreased the lag length, with a reduction under −20% compared to the negative control, except for compound **6** which decreased λ by −22.9%. A shorter lag phase allows for earlier divisions, enabling the cell to produce a higher number of progenies. This provides microorganisms with an ecological advantage in cases of competition for limited resources. Thus, the decrease of lag duration is generally considered beneficial, especially for populations in favorable conditions.^21^

In conclusion, obtained data suggested that molecules **14**, **25**, and **28** had a weak inhibitory effect on C. albicans growth; besides, no total biocidal activity was observed. Statistical analysis showed no differences between the growth parameters of C. albicans grown without (PBS) and with 3% DMSO (Table 2), indicating that the addition of the solvent to maintain the ZA-derivative in solution did not affect planktonic growth. Therefore, the differences found between **14**, **25**, and **28** and the controls were attributed to the sole effect of ZA-analogues.

### Adhesion Assay

The novel emerging antibiofilm strategies would involve compounds that interfere with key stages of biofilm formation, without killing biofilm-forming fungi, but rather by disarming them. Such approaches can play a crucial role in shaping the spread of antimicrobial resistance.^22^ A growing body of research has indicated that the transition of microorganisms from a planktonic to a biofilm state is one of the primary mechanisms that triggers C. albicans biofilm formation. Switching from yeast to hyphal growth not only allows C. albicans to develop a biofilm but also enables it to be highly invasive and escape macrophage engulfment.^23^ Indeed, the selection of compounds that avoid this primary mechanism, limiting the attachment of cells onto the substrates, and acting with mechanisms not affecting cell viability, is of considerable attention to prevent biofilm formation.^15^

One major mechanism of ZA against C. albicans is to reduce cell adhesion on both hydrophilic and hydrophobic surfaces, limiting its ability to switch between yeast and hyphal growth forms and reducing the capability to develop filamentous structures.^7^ Proteomic studies by Cattò et al.^9^ highlighted that when C. albicans was treated with ZA, the proteins Rbt1 and Ihd1 were downregulated. Rbt1 and Ihd1 are implicated in C. albicans adhesion and stable attachment of hyphae to the surface as they are linked to the core of filamentation formation, including germ tube formation followed by hyphal elongation.^24–29^

According to previous considerations, this research focused on the adhesion process of C. albicans biofilm formation, which was considered of major importance in comparing the antibiofilm activity of ZA with its derivatives. The ability of each ZA derivative to interfere with the first step of biofilm formation, specifically the adhesion of cells to the substrate, was analyzed using a microtiter assay. ZA analogues that affected cell adhesion by less than 20% compared to the negative control were considered to have no significant biological activity, those between 20% and 30% were considered to have low activity, those between 30% and 40% were considered to have moderate activity, and those with more than 40% were considered to have excellent activity.^12^ Data obtained from the adhesion assay and ANOVA statistical comparisons are reported in Tables 3 and S2, respectively.

**Table 3:** C. albicans Adhered Cells without (PBS and DMSO 3%) and with ZA-Analogues^a^

compound	n. adhered cells/cm^2^
PBS	(9.82 ± 1.70) × 10^5^ [−15.2]
DMSO 3%	(11.58 ± 2.61) × 10^5^ [0.0]
1 (ZA)	(3.61 ± 0.97) × 10^5^ [−68.8]*
6	(4.36 ± 1.01) × 10^5^ [−62.4]*
13	(4.66 ± 1.10) × 10^5^ [−59.8]*
19	(6.00 ± 1.81) × 10^5^ [−48.2]*
3	(6.03 ± 1.76) × 10^5^ [−47.9]*
31	(7.77 ± 0.76) × 10^5^ [−32.9]
16	(7.94 ± 2.00) × 10^5^ [−31.5]
18	(8.04 ± 0.94) × 10^5^ [−30.6]
7	(8.21 ± 1.29) × 10^5^ [−29.1]
5	(8.96 ± 2.66) × 10^5^ [−22.7]
30	(9.07 ± 1.50) × 10^5^ [−21.7]
2	(9.10 ± 2.67) × 10^5^ [−21.4]
8	(9.24 ± 0.85) × 10^5^ [−20.2]
10	(9.40 ± 2.31) × 10^5^ [−18.8]
15	(9.57 ± 2.83) × 10^5^ [−17.3]
25	(9.86 ± 1.05) × 10^5^ [−14.8]
14	(9.91 ± 2.22) × 10^5^ [−14.5]
29	(10.06 ± 2.60) × 10^5^ [−13.1]
4	(10.37 ± 2.83) × 10^5^ [−10.4]
21	(10.54 ± 2.70) × 10^5^ [−9.0]
24	(10.55 ± 0.80) × 10^5^ [−8.9]
9	(10.83 ± 1.59) × 10^5^ [−6.5]
23	(10.94 ± 1.08) × 10^5^ [−5.5]
28	(10.95 ± 3.74) × 10^5^ [−5.5]
27	(11.15 ± 3.08) × 10^5^ [−3.7]
17	(11.29 ± 2.14) × 10^5^ [−2.5]
26	(11.46 ± 1.17) × 10^5^ [−1.1]
22	(11.47 ± 2.33) × 10^5^ [−1.0]
12	(11.62 ± 3.96) × 10^5^ [0.3]
11	(13.33 ± 2.92) × 10^5^ [15.1]
20	(17.04 ± 0.88) × 10^5^ [47.2]*

a Data represent the mean ± standard deviation of at least three independent measurements. The values in the square brackets indicate the percentage increase/decrease compared to the negative control obtained with 3% DMSO. A star indicates statistically significant differences (Tukey’s HSD, *p* ≤ 0.05) between the value and the negative control with 3% DMSO.

Multidimensional scaling (MDS) analysis (Figure 3) based on the Bray–Curtis distance matrix (Table S3) provided a visual distribution of adhesion assay data in 2D-space. Adhesion data were clustered into three main groups: (i) molecules ZA, **3**, **6**, **13**, and **19**, which significantly reduced biofilm formation according to ANOVA analysis (Table S2) (group 1). Furthermore, these compounds clustered into three subsets: compound **1**, group **6** and **13**, and group **3** and **19**; (ii) molecule **20**, which significantly promoted biofilm formation, clustered alone (group 2); (iii) all the remaining molecules, which did not have a significant effect on cell adhesion, formed a separate and distant cluster (group 3).

**Figure 3:**
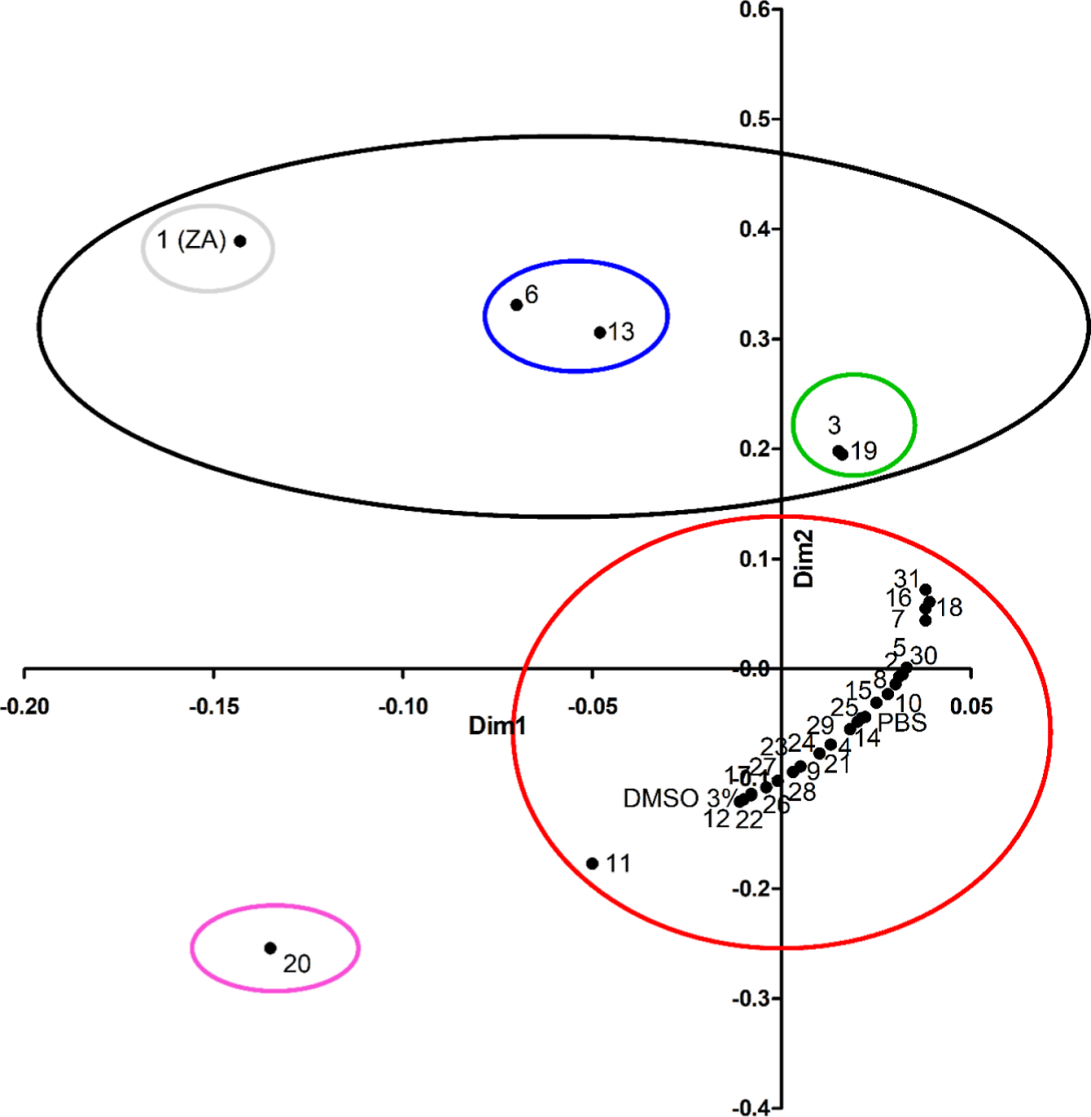
MDS analysis (stress: 0.08) based on Bray–Curtis distance showing a visual distribution of adhesion data between molecules. Molecules that significantly reduced biofilm formation are clustered in the black ring (group 1), with three subsets identifiable in gray, blue, and green rings. The molecule that promotes biofilm formation is located in the pink ring (group 2). Molecules that did not show a significant effect on cell adhesion are clustered in the red ring (group 3).

ZA and compounds **3**, **6**, **13**, and **19** in group 1 exhibited optimal antibiofilm activity by reducing biofilm formation by more than 47.8% (Tables 3 and S2). The Bray–Curtis distance matrix (Table S3) indicated that compound **6** had the antibiofilm activity most similar to ZA, followed by compound **13**. Statistical analysis revealed that the addition of the solvent did not contribute to the antiadhesion effect, as there were no differences in the number of adhered cells of C. albicans without (PBS) or with 3% DMSO (Table 3). Therefore, the antibiofilm activity of compounds **3**, **6**, **13**, and **19** was attributed solely to the effect of ZA-analogues. Furthermore, this activity was observed at sublethal concentrations, as these chemicals did not shift the C. albicans growth curve when the planktonic fungus was grown in their presence (Table 2). This is a promising perspective as it suggests that these compounds inhibit biofilm formation without increasing the risk of drug-resistant mutations as the selection pressure is avoided.^30^

Our results, combined with those from previous studies, demonstrated that compounds **3**, **13**, and **19** have a broad range of activities against fungal biofilms. Kimani et al.^31^ proved that compounds **3**, **13**, and **19** were effective against various food spoilage yeasts, e.g., Pichia anomala, Saccharomyces cerevisiae, Schizosaccharomyces pombe, and Debaryomyces hansenii, with biofilm inhibition effects ranging from 48% to 91%. Perez-Castillo et al.^32^ confirmed the activity of compound **3** and some of its derivatives against different Candida strains, showing that this compound can bind to multiple targets. Similarly, Kannan and Girija^33^ concluded that compound **3** is a good alternative for treating biofilm-related infections caused by five species of fluconazole-resistant Candida sp. Ferreira et al.^34^ reported that **13**-loaded liquid crystalline systems were highly effective against established biofilms of different Candida strains. Hu et al.^35^ showed that **13** inhibited anthracnose in plants caused by the fungus Colletotrichum gloeosporioides, while Sevinç and Özer^36^ demonstrated that **13** played an important role in the resistance of pumpkin seed to the adhesion of Fusarium proliferatum. Santiago et al.^37^ reported that molecule **19** affected teliospore germination, haploid sporidia production, and dikaryotic mycelium appearance in Sporisorium scitamineum on plant epidermal surface. Notably, compound **19** is a precursor of many biologically active secondary compounds that are crucial in plants’ defense mechanism against biofilm-related infection.^38^

Our study showed that compound **20** in group 2 promoted biofilm formation (Table 3). Carbon assay results revealed that molecule **20** was not a carbon and energy source for C. albicans. Therefore, the pro-biofilm activity versus C. albicans was not due to the compound acting as a growth substrate. It has been established that many secondary metabolites from plants, enhance biofilm formation at subinhibitory concentrations.^39^ Notably, compounds that alter biofilm pathways to promote formation show great promise for fostering beneficial biofilms.^39^ Research by Guzman^40^ reported different results, showing that compound **20** had strong inhibitory activity against both bacteria and fungi, with a greater effect on fungal species than bacteria. Wang et al.^41^ proved that **20** was a potential therapeutic drug against Aspergillus fumigatus, affecting fungal cell wall synthesis and membranes permeability. However, these studies differ from ours as they focused on microorganisms in planktonic life mode and tested compound efficacy at lethal concentrations. Biofilm formation is influenced by the external environment. The same molecules can either inhibit cell adhesion or promote cell aggregation, leading to biofilm development, depending on the concentration tested, following a hormetic response.^42^

Cinnamic acid derivatives have been reported to modulate quorum sensing in bacterial biofilms.^43^ To the best of the authors’ knowledge, similar information for fungal biofilms has not been reported in the literature. However, the authors speculate that cinnamic acid compounds that both inhibit and stimulate biofilm formation may be involved in some still unknown quorum sensing mechanisms.

Although molecules **14**, **25**, and **28** displayed weak inhibitory effects on planktonic growth, they did not exhibit biologically relevant activity in the biofilm form of life. Microbial cells in biofilms are physiologically distinct from planktonic cells of the same organism.^44^ Biofilms provide a protective barrier, making biofilm cells more resistant to chemicals than their planktonic counterpart.^45^ This may explain the discrepancy in results between planktonic and biofilm life modes.

Multivariate analysis of variance (MANOVA) coupled with *t*-test analysis (Tables S4 and S5) confirmed that certain structural features influence cell adhesion performance of each derivative (Figure 4).

**Figure 4:**
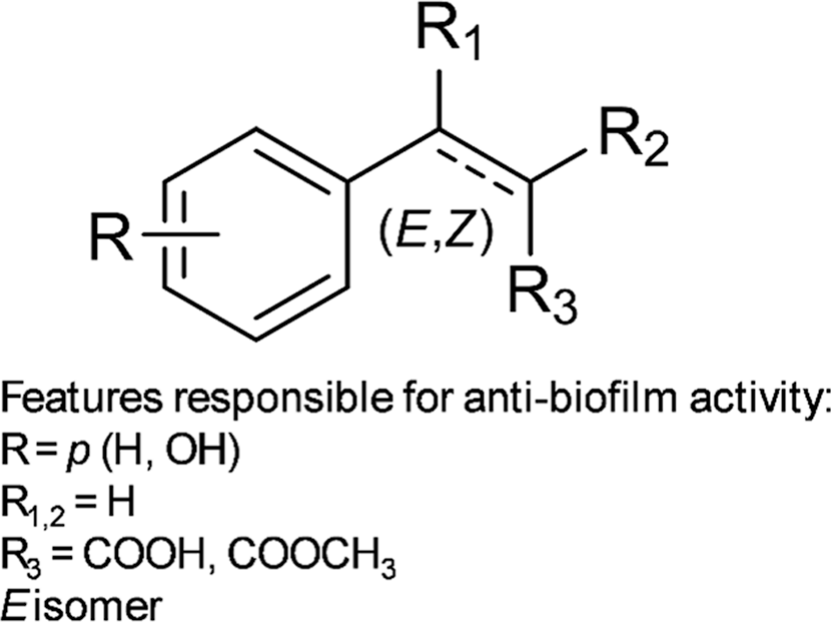
Cinnamic derivative features conferring the antibiofilm activity.

MANOVA results highlighted that independent variables, i.e., the presence of a double bond and the *cis*/*trans* configuration, alone and as interacting variables, had a significant impact on the dependent variable, i.e. cells adhesion (Table S4a–c). Specifically, the presence of the double bond in the side chain (**3** vs **5**, **13** vs **15**, Table S5a,b) with *trans* (*E*) configuration (ZA vs **2**, **3** vs **4**, **13** vs **14**, Table S5c–e) appeared to be crucial for the antibiofilm performance of the compounds.

MANOVA showed that both the function of the side chain and the presence or absence of a carboxylic function, considered as independent variables, both individually and in an interactive manner, were significantly correlated with the number of adhered cells (Table S4d). Indeed, a carboxylic function (**3** vs **10**, **3** vs **12**, Table S5f,g) without esterification (**3** vs **7** (*p* < 0.1), **13** vs **16**, except for **6** which showed activity comparable to **13**, Table S5h–j) ensured the antibiofilm effectiveness of the compounds.

MANOVA analysis showed that the presence of a substituent and its position on the phenyl ring, both alone and as interacting independent variables, significantly affected the number of cells (Table S4e,f). Indeed, statistical analysis highlighted that only the presence of the substituent in position 4 (*para*) influenced the number of adhered cells, whereas substituents in positions 2 (*ortho*) and 3 (*meta*) did not affect the dependent variable (Table S4g). Therefore, while the substitution on the phenyl ring at the *ortho* position had a detrimental effect (**3** vs **18**, **7** vs **9**, Table S5k,l), as did *meta* position (**3** vs **17**, **23**, Table S5m,n), the presence and nature of the substituents at the *para* position modulated the antibiofilm activity (**3** vs **13**, **20**, **22**, **27**, **28**, **29**, **30**, **31**, Tables S4h and S5o–v). The effect was not relevant for electron withdrawing groups (**22**, **29**, **28**, with the exception of **30** showing low activity), instead it led to a significant antibiofilm activity in the presence of some electron donor groups (**31** and **13**, with the exception of **20** causing enhanced adhesion), and in particular, the hydroxyl group conferred good antibiofilm activity (**3** vs **13**, Table S5o). Additionally, MANOVA analysis highlighted that substituents in the *para* position affected cell adhesion even when another substituent in the *ortho* or *meta* position was present on the phenyl ring (Table S4g).

Previous observations agree with the research of Kimani et al.^31^ and Adheboye et al.^46^ indicating that the inhibitory effects of some phenolic compounds of the same chemical family against the yeast Saccharomyces cerevisiae varied with the type of functional group attached to the phenyl ring. These authors showed that compound **3** had a more robust effect on the yeast than compounds **13** and **19** that have hydroxy groups substituted on the phenyl ring. Moreover, Camaioni et al.^47^ proved that there was an increase in the C. albicans biological activity of cinnamic acid derivatives when hydroxy groups were attached to the aromatic moiety and that the activity of the compounds decreased as their lipophilicity increased.

### Molecular Modeling Studies

As proposed by De Vita et al.,^48^ the cinnamic moiety seems to play a key role in the antibiofilm properties of certain cinnamic derivatives. Notably, in many examples, the ester or amide derivatives synthesized from nonbiofilm-active amines or alcohols displayed significant activity against biofilms.^48^ However, the molecular target, as well as the molecular mechanism of the antibiofilm activity of these derivatives, remains unclear. It is reasonable to think that they can modulate the redox balance of the cellular environment, by reacting with species like H_2_O_2_ or other reactive oxygen species or by modulating the activity of enzymes with oxidoreductase catalytic activity. In our previous papers,^12^ we have already demonstrated that ZA, **3**, and **19** are able to influence the catalytic activity of the E. coli WrbA enzyme, a NADP­(H) quinone oxidoreductase involved in biofilm formation. Therefore, to elucidate the mechanism of action of the compounds investigated here, we built a homology model of the C. albicans NADP­(H) quinone oxidoreductase more strictly related to the E. coli WrbA (see Experimental Section for details), assuming that the antibiofilm mechanism of this series of compounds is conserved from prokaryotic to eukaryotic cells. The model generated by the SwissModel server was equilibrated in water by 500 ns of molecular dynamics (MD) simulations, then docking and MD simulations were accomplished to predict the binding mode of the ZA analogues displaying the highest antibiofilm activity, such as ZA, **3**, **13**, and **19**. Compounds **6** was excluded from these calculations, because it is highly probable that it is rapidly converted to the free acid (**3**) by the presence of esterases in the extra- or intracellular environment.

The computational results suggested that all compounds are bound to the surface of flavin mononucleotide (FMN), stabilized by π–π stacking contacts with it. The OH groups of compounds **13** and **19** did not seem to be involved in any interactions with the biological counterpart, while the acidic moieties are bound by H-bonds with Tyr145­(B), His135­(C), Thr117­(D), and Ala151­(B), the latter by a water molecule bridge. These contacts are further stabilized by a π–π stacking between the phenyl ring of the ligands and the side chain of Trp99­(C) (Figure 5). These interactions were stable over three replicas of 250 ns-long MD simulations for all compounds except for ZA. In fact, this ligand unbound from the binding site after early steps of MD simulations in each replica. This suggests that ZA may not fit well into the enzyme’s binding site, indicating that its antibiofilm activity could be driven by alternative or multiple mechanisms. On the other hand, under experimental conditions, ZA might be converted into compound **13** through the action of fungal sulfatases.

**Figure 5:**
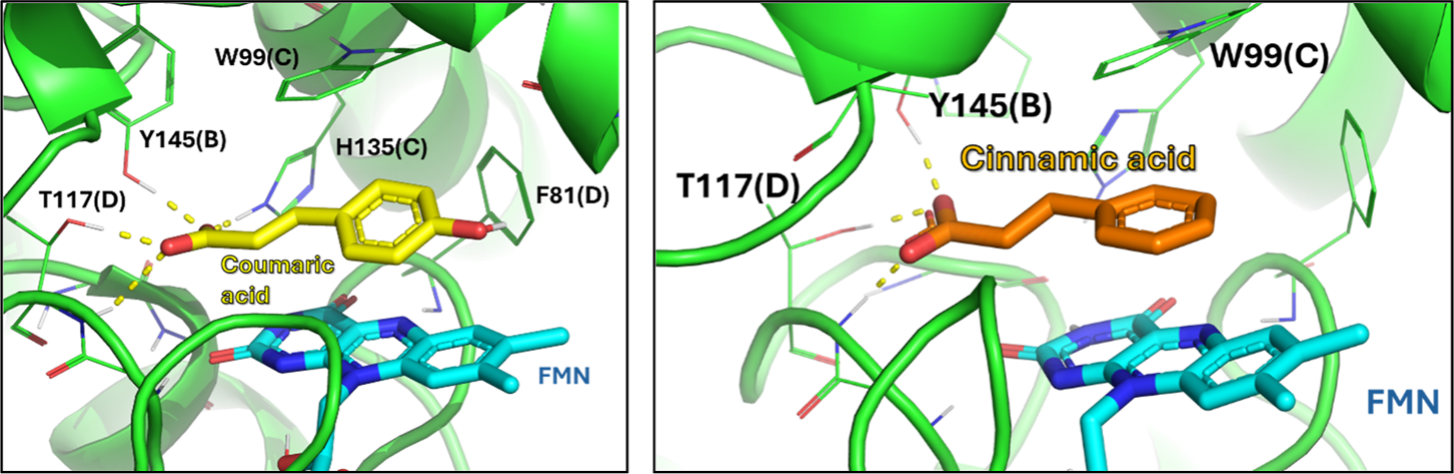
Binding mode predicted for **13** (yellow on the left) and **3** (orange on the right) as resulted from computational studies (docking and MD simulations). The H-bonds are depicted by dashed yellow lines.

Finally, it is important to stress that given the plethora of enzymes governing the biofilm machinery, it is inherently challenging to identify the specific enzyme(s) capable of binding our compounds. The complexity of cellular enzymatic networks means that these computational studies can only provide a starting point for further investigation rather than a definitive answer.

## Conclusions

Among the analyzed ZA-analogues, compounds **3**, **6**, **13**, and **19** emerged as the most relevant compounds against C. albicans biofilm formation, noteworthy without toxic impact on the fungal life. This is an important result in the current era where the rapid emergence of resistant strains has become a global major problem, with only three classes of antifungals currently available.^49^ In contrast, compound **20** promoted cell adhesion at the tested concentration, suggesting that it could be used to promote biofilm growth for biotechnological applications.

The results reported here confirmed different antibiofilm activity of ZA-analogues against C. albicans depending on their chemical structure. The position and nature of the functional group linked to the phenyl ring, the presence of a *cis*/*trans* double bond configuration, and the function of the side chain all seem to affect biofilm development. A double bond in *trans* configuration (**3**, **6**, **13**, and **19**), along with a free carboxylic group in the side chain (**3**, **13**, and **19**), and the *para* substitution with a hydroxyl group (**13**, **19**) guaranteed the antibiofilm activity of the molecules. This is of relevant importance for developing new ZA-based technologies and implementing any molecule-based applications and materials against fungal biofilms.

Among the most interesting compounds (**6** and **13**), the natural derivative **13**, which possesses the highlighted features, showed an activity comparable to the reference ZA (with the added advantage of being commercially available). In this regard, it can be assumed that in the assay conditions, ZA undergoes a hydrolysis reaction leading to the corresponding *p*-hydroxyl derivative. Molecular docking studies performed on **13**, considering ZA and **3** as references, suggested its interaction with NADP­(H) quinone oxidoreductase and, therefore, its possible involvement in the microorganisms’ redox balance.

## Experimental Section

### Synthesis of Cinnamic Acid Derivatives

ZAs (*E* and *Z*, **1** and **2** respectively), cinnamic acid (*Z*, **4**), and some compounds (**6**, **27** and **28**) were synthesized starting from the appropriate isomer (or their geometries were confirmed by proton–proton coupling constants) through slightly modified literature procedures,^12^ while the others (**3**, **5**, **7**–**26**, **29**–**31**) were purchased from Merck and directly used without further purification.

### C. albicans Strain and Growth Conditions

The fungal model C. albicans ATCC MYA-2876 was used in the study. The strain was stored at −80 °C in a freezer medium containing 2% peptone and 20% glycerol. The microorganism was grown at 30 °C for 24 h in the yeast nitrogen base broth supplemented with 50 mM of glucose (YNBg, Sigma-Aldrich, St. Louis, USA) and fungal cells were washed three times in Phosphate Buffer Saline (PBS, Sigma-Aldrich) before being used in the subsequent experiments.

### ZA-Analogues as Carbon and Energy Source

The ability of C. albicans to grow with each ZA-derivative as the sole carbon and energy source was tested by incubating 10^6^ cells/mL in a mineral medium (KH_2_PO_4_ 30 g/L, Na_2_HPO_4_ 70 g/L, NH_4_Cl 10 g/L, pH 7) supplemented with 35 μM of each molecule and 3% DMSO. Fungi were incubated at 30 °C for 72 h, and growth was followed by measuring optical density at 600 nm (OD600) at the end. The positive control consisted of the mineral medium supplemented with glucose at concentrations of both 35 μM and 10 mM. Each experiment was repeated three times.

### Planktonic Growth in the Presence of ZA-Analogues

Planktonic growth of fungi in the presence of each ZA-analogue was performed in 96-well microtiter plates according to Cattò et al.^12^ Briefly, a washed overnight culture of fungi was added to YNBg supplemented with 35 μM of each ZA-analogue and 3% DMSO to reach the final concentration of 10^6^ cells/mL. Fungi were grown at 30 °C, and the OD600 was measured every 15 min for 48 h using the Infinite 200 PRO Microplate Reader (Tecan, Männedorf, Switzerland). Positive and negative controls were grown by inoculating or not inoculating C. albicans cells in YNBg or YNBg supplemented with 3% DMSO, respectively. The OD600 of the culture suspensions minus the OD600 of the noninoculated medium was plotted against the incubation time, and the absorbance-based growth kinetics were constructed for each ZA-derivative using GraphPad Prism software (version 5.0, San Diego, USA). The polynomial Gompertz model was used to fit the growth curves. The Lag phase length (λ), maximum exponential growth rate (μ_m_), and end point of kinetic growth (Y_M_) were calculated for each condition using GraphPad Prism software.^50,51^ ZA-analogues were considered to affect planktonic growth when an increase in λ and a decrease in μ_m_ and Y_M_ were observed. Three biological replicates were performed for each molecule, and at least four technical replicates were performed for each experiment.

### Adhesion Assay

The adhesion assay was performed as previously described by Glasenapp et al.^52^ Briefly, 200 μL of PBS containing 10^6^ cells, with the addition of 35 μM of each ZA-derivative and 3% DMSO, were placed in hydrophobic black-sided microtiter plate wells and were incubated for 24 h at 30 °C. The wells were then washed twice with 200 μL of PBS, and the adhered cells were stained with 0.1 mg/mL Fluorescent Brightener 28 (Sigma-Aldrich, USA) for 20 min in the dark at room temperature. Experiments were also carried out with PBS and 3% DMSO as negative controls. The fluorescence intensity was measured using the Infinite 200 PRO Microplate Reader with 335 nm excitation and 433 nm emission wavelengths. The number of adhered cells was calculated by using a standard curve of fluorescence intensity. Data were normalized to the area, and the means were calculated. Three biological replicates were performed for each molecule, with at least four technical replicates performed for each experiment.

### Statistical Analysis

The percentage of reduction/increase in planktonic growth or cell adhesion compared to the negative control prepared with 3% DMSO was calculated as (ZA-derivative data – negative control data) × 100/negative control data.

A two-tailed ANOVA analysis was applied to statistically evaluate any significant differences among the samples. This analysis was performed after verifying data independence (Pearson’s Chi-square test), normal distribution (D’Agostino-Pearson normality test), and homogeneity of variances (Bartlett’s test). Post hoc Tukey’s HSD test was used for pairwise comparisons to determine the significance of the data. When a comparison between two samples was necessary, a student’s *t*-test was also applied. Bray–Curtis distance scores among adhesion assay data were calculated, and distances were plotted using MDS analysis. To evaluate the correlation between C. albicans cellular adhesion and the type and position of substituents on the phenyl ring, presence of the double bond, *E*/*Z* isomers, and side chain modifications, a MANOVA was performed. Statistically significant results were determined by *p*-values ≤0.05. All statistical analyses were performed using the XLSTAT package (XLSTAT 2019.3.2).

### Molecular Modeling

Since the structure of C. albicans NAD­(P)H quinone oxidoreductase has never been obtained by X-ray or NMR studies, a homology model based on the E. coli WrbA crystal structure was generated. Among the numerous structures of E. coli WrbA available in the Protein Data Bank (PDB), it was selected an X-ray structure of a protein tetramer in complex with benzoquinone (BQ) (PDB accession code 4YQE) as the template, since the FMN binding site is shared by at least three monomers.^53^ On the other hand, four sequences of C. albicans flavodoxin-like quinone oxidoreductases are deposited in the UNIPROT database. To choose the one more closely related to the prokaryotic form, they were aligned to the sequence of E. coli (Uniprot code P0A8G6), in order to find the one with the highest homology, by using the ClustalW Web server (https://www.genome.jp/tools-bin/clustalw). The alignment suggested that the NAD­(P)H quinone oxidoreductase PST2 (Uniprot code Q59Y37) exhibited the highest identity percentage (41.4%) while having a sequence length only 3 residues longer than WrbA. Consequently, the C. albicans NAD­(P)H quinone oxidoreductase homology model was constructed by SWISS-MODEL server and the quality of this model was evaluated by analyzing the secondary structure of the protein using the Ramachandran plot, which combines the Φ/Ψ dihedral angles of each amino acid (Figure S1). Once some divergences were amended using the “Protein Preparation Tool” of Maestro (release 2024-2, Schrödinger, LLC, New York, USA), the initial model was energy minimized and equilibrated in water by MD simulations using the Maestro (Schrödinger, LLC, New York, USA) standard protocol. Here, once assigned the protonation state of the protein residues at pH 7.4, and the OPLS4 force field were assigned, a cubic box of water molecules (almost 18,500, represented by the TIP3P model) was built around the enzyme tetramer and energy minimized by the Desmond algorithm implemented in Maestro.^54–56^ A single run of 500 ns MD simulations was accomplished, and the “Simulation Interactions Diagram” tool was employed to evaluate the stability of the simulating system. Then, docking calculations of the compounds displaying the highest antibiofilm activity (ZA, **3**, **13**) were accomplished using the enzyme model resulting at the end of MD simulations. The Glide algorithm was employed to this aim, using as docking site the one identified by the presence of BQ in the reductase site of WrbA.^57^ In fact, it is known that the natural ligand (BQ) is positioned on the flavin-mononucleotide (FMN) rings in order to be reduced by the enzyme and the involvement of the NADP­(H). The best docking pose of each compound was simulated in complex with the enzyme by 500 ns long MD simulations, using the same protocol applied for the enzyme in the *apo* state. Then, the “Simulation Interactions Diagram” tool of Maestro was employed to evaluate the stability of the ligand in the reductase catalytic site.

## Supplementary Material



## Notes

C. albicans planktonic growth with ZA-analogues as sole source of carbon and energy; ANOVA analysis applied to statistically evaluate any significant differences among C. albicans adhesion data; Bray–Curtis proximity matrix obtained from averages of ZA-analogues adhesion data; MANOVA analysis obtained from averages of ZA-analogues adhesion data; Student’s *t*-test analysis performed to compared adhesion data of some relevant ZA-analogues; and Ramachandran plot of the C. albicans NAD­(P)H quinone oxidoreductase homology models (PDF)
