# Supplementary material for: Insights on Zosteric Acid Analogues Activity Against Candida albicans Biofilm Formation
Source: ACS Omega. 2025 May 20;10(21):22285–95. doi: 10.1021/acsomega.5c03581 (PMC12138599; doi:10.1021/acsomega.5c03581)

Supporting Information

Insights on zosteric acid analogues activity against *Candida albicans* biofilm formation

Cristina Cattò[a], Enrico M. A. Fassi[b], Giovanni Grazioso[b], Arianna Gelain[b], Stefania Villa[b], Francesca Cappitelli[a]*

[a]University of Milan, Department of Food, Environmental and Nutritional Sciences, via G. Celoria 2, 20133 Milan, Italy

[b]University of Milan, Department of Pharmaceutical Sciences, via L. Mangiagalli 25, 20133 Milan, Italy

*corresponding author, cristina.catto@unimi.it

Table S1. *C. albicans* planktonic growth with ZA-analogues as sole source of carbon and energy. Data report the mean ± standard deviation of OD600 values taken after 72 h of incubation and comes from at least three independent measurements.

**Compound**	**OD600**
Glucose 35 µM	0.035 ± 0.009
Glucose 10 mM	0.358 ± 0.055
1 (ZA)	0.037 ± 0.005
2	0.058 ± 0.027
3	0.033 ± 0.002
4	0.009 ± 0.001
5	0.032 ± 0.007
6	0.033 ± 0.001
7	0.035 ± 0.003
8	0.038 ± 0.004
9	0.024 ± 0.006
10	0.025 ± 0.002
11	0.026 ± 0.001
12	0.037 ± 0.008
13	0.033 ± 0.003
14	0.034 ± 0.002
15	0.025 ± 0.008
16	0.029 ± 0.005
17	0.034 ± 0.004
18	0.038 ± 0.004
19	0.024 ± 0.022
20	0.026 ± 0.002
21	0.035 ± 0.004
22	0.033 ± 0.003
23	0.034 ± 0.013
24	0.033 ± 0.003
25	0.039 ± 0.003
26	0.032 ± 0.003
27	0.032 ± 0.015
28	0.009 ± 0.003
29	0.028 ± 0.010
30	0.028 ± 0.002
31	0.030 ± 0.005

Table S2. ANOVA analysis applied to statistically evaluate any significant differences among *C. albicans* adhesion data (Table S2a). Post hoc Tukey's honestly significant difference (HSD) test was used for pairwise comparisons to determine the significance of the data (Table S2b). Different letters indicate significant differences between the means of different coatings (Tukey's HSD) (Table S2c). Statistically significant results were decided by p-values ≤ 0.05. p-value signification codes: 0 < *** ≤ 0.001 < ** ≤ 0.01 < * ≤ 0.05 < ° ≤ 0.1.

Table S2a

Source	DF	Sum of squares	Mean squares	F	Pr > F	p-values signification codes
Model	32.000	17963977704451.000	561374303264.093	8.595	**<0.0001**	***
Error	375.000	24492636763200.400	65313698035.201			
Corrected Total	407.000	42456614467651.400				

Table S2b

Contrast	Difference	Standardized difference	Critical value	Pr > Diff	p-values codes	Significant (p≤0.05)	Lower bound (95%)	Upper bound (95%)
PBS vs ZA	621179.626	4.282	3.826	0.010	**	Yes	66088.032	1176271.220
PBS vs 8	58221.080	0.401	3.826	1.000		No	-496870.513	613312.674
PBS vs 7	161445.774	1.113	3.826	1.000		No	-393645.820	716537.367
PBS vs 6	546573.203	3.767	3.826	0.061	°	No	-8518.391	1101664.796
PBS vs 5	86584.233	0.597	3.826	1.000		No	-468507.361	641675.827
PBS vs 31	205097.972	1.414	3.826	1.000		No	-349993.622	760189.565
PBS vs 30	75526.441	0.521	3.826	1.000		No	-479565.153	630618.035
PBS vs 3	378960.850	2.612	3.826	0.742		No	-176130.744	934052.444
PBS vs 2	71943.824	0.496	3.826	1.000		No	-483147.770	627035.418
PBS vs 19	382824.418	2.639	3.826	0.722		No	-172267.175	937916.012
PBS vs 18	178331.406	1.229	3.826	1.000		No	-376760.188	733423.000
PBS vs 16	188659.208	1.300	3.826	1.000		No	-366432.386	743750.802
PBS vs 15	24915.949	0.172	3.826	1.000		No	-530175.645	580007.543
PBS vs 13	516785.008	3.562	3.826	0.114		No	-38306.586	1071876.602
PBS vs 10	42109.701	0.290	3.826	1.000		No	-512981.893	597201.295
DMSO 3% vs ZA	796960.970	5.493	3.826	<0.0001	***	Yes	241869.377	1352052.564
DMSO 3% vs PBS	175781.345	1.212	3.826	1.000		No	-379310.249	730872.938
DMSO 3% vs 9	75248.472	0.519	3.826	1.000		No	-479843.122	630340.066
DMSO 3% vs 8	234002.425	1.613	3.826	1.000		No	-321089.169	789094.019
DMSO 3% vs 7	337227.118	2.324	3.826	0.908		No	-217864.476	892318.712
DMSO 3% vs 6	722354.547	4.979	3.826	0.000	***	Yes	167262.953	1277446.141
DMSO 3% vs 5	262365.578	1.808	3.826	0.997		No	-292726.016	817457.171
DMSO 3% vs 4	120966.657	0.834	3.826	1.000		No	-434124.937	676058.250
DMSO 3% vs 31	380879.316	2.625	3.826	0.732		No	-174212.278	935970.910
DMSO 3% vs 30	251307.785	1.732	3.826	0.999		No	-303783.808	806399.379
DMSO 3% vs 3	554742.194	3.824	3.826	0.050	*	Yes	-349.400	1109833.788
DMSO 3% vs 29	151742.215	1.046	3.826	1.000		No	-403349.379	706833.809

DMSO 3% vs 28	63411.097	0.437	3.826	1.000		No	-491680.496	618502.691
DMSO 3% vs 27	43186.698	0.298	3.826	1.000		No	-511904.896	598278.292
DMSO 3% vs 26	12582.248	0.087	3.826	1.000		No	-542509.346	567673.842
DMSO 3% vs 25	171919.081	1.185	3.826	1.000		No	-383172.513	727010.675
DMSO 3% vs 24	102636.714	0.707	3.826	1.000		No	-452454.880	657728.308
DMSO 3% vs 23	63973.281	0.441	3.826	1.000		No	-491118.313	619064.875
DMSO 3% vs 22	11443.095	0.079	3.826	1.000		No	-543648.499	566534.689
DMSO 3% vs 21	104158.375	0.718	3.826	1.000		No	-450933.218	659249.969
DMSO 3% vs 2	247725.168	1.707	3.826	0.999		No	-307366.425	802816.762
DMSO 3% vs 19	558605.763	3.850	3.826	0.046	*	Yes	3514.169	1113697.357
DMSO 3% vs 18	354112.751	2.441	3.826	0.852		No	-200978.843	909204.344
DMSO 3% vs 17	29107.262	0.201	3.826	1.000		No	-525984.332	584198.856
DMSO 3% vs 16	364440.553	2.512	3.826	0.810		No	-190651.041	919532.146
DMSO 3% vs 15	200697.293	1.383	3.826	1.000		No	-354394.300	755788.887
DMSO 3% vs 14	167434.518	1.154	3.826	1.000		No	-387657.076	722526.112
DMSO 3% vs 13	692566.352	4.774	3.826	0.001	**	Yes	137474.759	1247657.946
DMSO 3% vs 10	217891.045	1.502	3.826	1.000		No	-337200.548	772982.639
9 vs ZA	721712.498	4.975	3.826	0.000	***	Yes	166620.905	1276804.092
9 vs PBS	100532.872	0.693	3.826	1.000		No	-454558.721	655624.466
9 vs 8	158753.953	1.094	3.826	1.000		No	-396337.641	713845.546
9 vs 7	261978.646	1.806	3.826	0.997		No	-293112.948	817070.240
9 vs 6	647106.075	4.460	3.826	0.005	**	Yes	92014.481	1202197.669
9 vs 5	187117.105	1.290	3.826	1.000		No	-367974.488	742208.699
9 vs 4	45718.185	0.315	3.826	1.000		No	-509373.409	600809.778
9 vs 31	305630.844	2.107	3.826	0.971		No	-249460.750	860722.438
9 vs 30	176059.313	1.214	3.826	1.000		No	-379032.280	731150.907
9 vs 3	479493.722	3.305	3.826	0.226		No	-75597.872	1034585.316
9 vs 29	76493.743	0.527	3.826	1.000		No	-478597.851	631585.337
9 vs 25	96670.609	0.666	3.826	1.000		No	-458420.985	651762.203
9 vs 24	27388.242	0.189	3.826	1.000		No	-527703.352	582479.835
9 vs 21	28909.903	0.199	3.826	1.000		No	-526181.691	584001.497
9 vs 2	172476.696	1.189	3.826	1.000		No	-382614.897	727568.290
9 vs 19	483357.291	3.332	3.826	0.211		No	-71734.303	1038448.885
9 vs 18	278864.278	1.922	3.826	0.992		No	-276227.315	833955.872
9 vs 16	289192.080	1.993	3.826	0.986		No	-265899.513	844283.674
9 vs 15	125448.821	0.865	3.826	1.000		No	-429642.772	680540.415
9 vs 14	92186.046	0.635	3.826	1.000		No	-462905.548	647277.639
9 vs 13	617317.880	4.255	3.826	0.011	*	Yes	62226.287	1172409.474
9 vs 10	142642.573	0.983	3.826	1.000		No	-412449.021	697734.167
8 vs ZA	562958.546	3.880	3.826	0.042	*	Yes	7866.952	1118050.139
8 vs 7	103224.693	0.711	3.826	1.000		No	-451866.901	658316.287
8 vs 6	488352.122	3.366	3.826	0.194		No	-66739.471	1043443.716
8 vs 5	28363.153	0.195	3.826	1.000		No	-526728.441	583454.747
8 vs 31	146876.891	1.012	3.826	1.000		No	-408214.702	701968.485

8 vs 30	17305.361	0.119	3.826	1.000		No	-537786.233	572396.954
8 vs 3	320739.769	2.211	3.826	0.947		No	-234351.824	875831.363
8 vs 2	13722.744	0.095	3.826	1.000		No	-541368.850	568814.337
8 vs 19	324603.338	2.237	3.826	0.939		No	-230488.256	879694.932
8 vs 18	120110.326	0.828	3.826	1.000		No	-434981.268	675201.920
8 vs 16	130438.128	0.899	3.826	1.000		No	-424653.466	685529.722
8 vs 13	458563.928	3.161	3.826	0.314		No	-96527.666	1013655.521
7 vs ZA	459733.852	3.169	3.826	0.308		No	-95357.741	1014825.446
7 vs 6	385127.429	2.655	3.826	0.710		No	-169964.165	940219.023
7 vs 31	43652.198	0.301	3.826	1.000		No	-511439.396	598743.792
7 vs 3	217515.076	1.499	3.826	1.000		No	-337576.518	772606.670
7 vs 19	221378.645	1.526	3.826	1.000		No	-333712.949	776470.239
7 vs 18	16885.633	0.116	3.826	1.000		No	-538205.961	571977.226
7 vs 16	27213.435	0.188	3.826	1.000		No	-527878.159	582305.028
7 vs 13	355339.234	2.449	3.826	0.847		No	-199752.359	910430.828
6 vs ZA	74606.423	0.514	3.826	1.000		No	-480485.171	629698.017
5 vs ZA	534595.393	3.685	3.826	0.079	°	No	-20496.201	1089686.987
5 vs 7	74861.540	0.516	3.826	1.000		No	-480230.053	629953.134
5 vs 6	459988.970	3.171	3.826	0.307		No	-95102.624	1015080.563
5 vs 31	118513.739	0.817	3.826	1.000		No	-436577.855	673605.332
5 vs 3	292376.617	2.015	3.826	0.984		No	-262714.977	847468.210
5 vs 19	296240.185	2.042	3.826	0.981		No	-258851.408	851331.779
5 vs 18	91747.173	0.632	3.826	1.000		No	-463344.421	646838.767
5 vs 16	102074.975	0.704	3.826	1.000		No	-453016.619	657166.569
5 vs 13	430200.775	2.965	3.826	0.459		No	-124890.819	985292.369
4 vs ZA	675994.314	4.659	3.826	0.002	**	Yes	120902.720	1231085.908
4 vs PBS	54814.688	0.378	3.826	1.000		No	-500276.906	609906.282
4 vs 8	113035.768	0.779	3.826	1.000		No	-442055.826	668127.362
4 vs 7	216260.461	1.491	3.826	1.000		No	-338831.132	771352.055
4 vs 6	601387.890	4.145	3.826	0.016	*	Yes	46296.297	1156479.484
4 vs 5	141398.921	0.975	3.826	1.000		No	-413692.673	696490.515
4 vs 31	259912.659	1.792	3.826	0.997		No	-295178.934	815004.253
4 vs 30	130341.129	0.898	3.826	1.000		No	-424750.465	685432.723
4 vs 3	433775.538	2.990	3.826	0.440		No	-121316.056	988867.131
4 vs 29	30775.558	0.212	3.826	1.000		No	-524316.035	585867.152
4 vs 25	50952.424	0.351	3.826	1.000		No	-504139.169	606044.018
4 vs 2	126758.512	0.874	3.826	1.000		No	-428333.082	681850.106
4 vs 19	437639.106	3.017	3.826	0.419		No	-117452.488	992730.700
4 vs 18	233146.094	1.607	3.826	1.000		No	-321945.500	788237.688
4 vs 16	243473.896	1.678	3.826	0.999		No	-311617.698	798565.490
4 vs 15	79730.637	0.550	3.826	1.000		No	-475360.957	634822.231
4 vs 14	46467.861	0.320	3.826	1.000		No	-508623.733	601559.455
4 vs 13	571599.696	3.940	3.826	0.034	*	Yes	16508.102	1126691.290
4 vs 10	96924.389	0.668	3.826	1.000		No	-458167.205	652015.982

31 vs ZA	416081.654	2.868	3.826	0.538		No	-139009.939	971173.248
31 vs 6	341475.231	2.354	3.826	0.895		No	-213616.363	896566.825
31 vs 3	173862.878	1.198	3.826	1.000		No	-381228.716	728954.472
31 vs 19	177726.447	1.225	3.826	1.000		No	-377365.147	732818.041
31 vs 13	311687.036	2.148	3.826	0.962		No	-243404.557	866778.630
30 vs ZA	545653.185	3.761	3.826	0.062	°	No	-9438.409	1100744.779
30 vs 7	85919.333	0.592	3.826	1.000		No	-469172.261	641010.926
30 vs 6	471046.762	3.247	3.826	0.259		No	-84044.832	1026138.356
30 vs 5	11057.792	0.076	3.826	1.000		No	-544033.802	566149.386
30 vs 31	129571.531	0.893	3.826	1.000		No	-425520.063	684663.124
30 vs 3	303434.409	2.091	3.826	0.973		No	-251657.185	858526.003
30 vs 19	307297.977	2.118	3.826	0.969		No	-247793.616	862389.571
30 vs 18	102804.965	0.709	3.826	1.000		No	-452286.629	657896.559
30 vs 16	113132.767	0.780	3.826	1.000		No	-441958.827	668224.361
30 vs 13	441258.567	3.041	3.826	0.400		No	-113833.027	996350.161
3 vs ZA	242218.776	1.670	3.826	0.999		No	-312872.818	797310.370
3 vs 6	167612.353	1.155	3.826	1.000		No	-387479.241	722703.947
3 vs 19	3863.569	0.027	3.826	1.000		No	-551228.025	558955.162
3 vs 13	137824.158	0.950	3.826	1.000		No	-417267.436	692915.752
29 vs ZA	645218.755	4.447	3.826	0.005	**	Yes	90127.162	1200310.349
29 vs PBS	24039.129	0.166	3.826	1.000		No	-531052.464	579130.723
29 vs 8	82260.210	0.567	3.826	1.000		No	-472831.384	637351.803
29 vs 7	185484.903	1.278	3.826	1.000		No	-369606.691	740576.497
29 vs 6	570612.332	3.933	3.826	0.035	*	Yes	15520.738	1125703.926
29 vs 5	110623.362	0.762	3.826	1.000		No	-444468.231	665714.956
29 vs 31	229137.101	1.579	3.826	1.000		No	-325954.493	784228.695
29 vs 30	99565.570	0.686	3.826	1.000		No	-455526.023	654657.164
29 vs 3	402999.979	2.778	3.826	0.612		No	-152091.615	958091.573
29 vs 25	20176.866	0.139	3.826	1.000		No	-534914.728	575268.460
29 vs 2	95982.953	0.662	3.826	1.000		No	-459108.640	651074.547
29 vs 19	406863.548	2.804	3.826	0.591		No	-148228.046	961955.142
29 vs 18	202370.535	1.395	3.826	1.000		No	-352721.058	757462.129
29 vs 16	212698.338	1.466	3.826	1.000		No	-342393.256	767789.931
29 vs 15	48955.078	0.337	3.826	1.000		No	-506136.515	604046.672
29 vs 14	15692.303	0.108	3.826	1.000		No	-539399.291	570783.896
29 vs 13	540824.137	3.728	3.826	0.069	°	No	-14267.456	1095915.731
29 vs 10	66148.830	0.456	3.826	1.000		No	-488942.763	621240.424
28 vs ZA	733549.873	5.056	3.826	0.000	***	Yes	178458.279	1288641.467
28 vs PBS	112370.247	0.775	3.826	1.000		No	-442721.347	667461.841
28 vs 9	11837.375	0.082	3.826	1.000		No	-543254.219	566928.969
28 vs 8	170591.328	1.176	3.826	1.000		No	-384500.266	725682.921
28 vs 7	273816.021	1.887	3.826	0.994		No	-281275.573	828907.615
28 vs 6	658943.450	4.542	3.826	0.003	**	Yes	103851.856	1214035.044
28 vs 5	198954.480	1.371	3.826	1.000		No	-356137.113	754046.074

28 vs 4	57555.559	0.397	3.826	1.000		No	-497536.034	612647.153
28 vs 31	317468.219	2.188	3.826	0.953		No	-237623.375	872559.813
28 vs 30	187896.688	1.295	3.826	1.000		No	-367194.906	742988.282
28 vs 3	491331.097	3.387	3.826	0.184		No	-63760.497	1046422.691
28 vs 29	88331.118	0.609	3.826	1.000		No	-466760.476	643422.712
28 vs 25	108507.984	0.748	3.826	1.000		No	-446583.610	663599.578
28 vs 24	39225.617	0.270	3.826	1.000		No	-515865.977	594317.210
28 vs 23	562.184	0.004	3.826	1.000		No	-554529.410	555653.777
28 vs 21	40747.278	0.281	3.826	1.000		No	-514344.316	595838.872
28 vs 2	184314.071	1.270	3.826	1.000		No	-370777.523	739405.665
28 vs 19	495194.666	3.413	3.826	0.171		No	-59896.928	1050286.259
28 vs 18	290701.653	2.004	3.826	0.985		No	-264389.940	845793.247
28 vs 16	301029.455	2.075	3.826	0.976		No	-254062.138	856121.049
28 vs 15	137286.196	0.946	3.826	1.000		No	-417805.398	692377.790
28 vs 14	104023.421	0.717	3.826	1.000		No	-451068.173	659115.014
28 vs 13	629155.255	4.337	3.826	0.008	**	Yes	74063.661	1184246.849
28 vs 10	154479.948	1.065	3.826	1.000		No	-400611.646	709571.542
27 vs ZA	753774.272	5.196	3.826	0.000	***	Yes	198682.679	1308865.866
27 vs PBS	132594.646	0.914	3.826	1.000		No	-422496.947	687686.240
27 vs 9	32061.774	0.221	3.826	1.000		No	-523029.820	587153.368
27 vs 8	190815.727	1.315	3.826	1.000		No	-364275.867	745907.321
27 vs 7	294040.420	2.027	3.826	0.983		No	-261051.174	849132.014
27 vs 6	679167.849	4.681	3.826	0.002	**	Yes	124076.255	1234259.443
27 vs 5	219178.880	1.511	3.826	1.000		No	-335912.714	774270.473
27 vs 4	77779.959	0.536	3.826	1.000		No	-477311.635	632871.552
27 vs 31	337692.618	2.328	3.826	0.907		No	-217398.976	892784.212
27 vs 30	208121.087	1.435	3.826	1.000		No	-346970.506	763212.681
27 vs 3	511555.496	3.526	3.826	0.126		No	-43536.098	1066647.090
27 vs 29	108555.517	0.748	3.826	1.000		No	-446536.077	663647.111
27 vs 28	20224.399	0.139	3.826	1.000		No	-534867.195	575315.993
27 vs 25	128732.383	0.887	3.826	1.000		No	-426359.211	683823.977
27 vs 24	59450.016	0.410	3.826	1.000		No	-495641.578	614541.610
27 vs 23	20786.583	0.143	3.826	1.000		No	-534305.011	575878.177
27 vs 21	60971.677	0.420	3.826	1.000		No	-494119.917	616063.271
27 vs 2	204538.470	1.410	3.826	1.000		No	-350553.123	759630.064
27 vs 19	515419.065	3.553	3.826	0.117		No	-39672.529	1070510.659
27 vs 18	310926.053	2.143	3.826	0.964		No	-244165.541	866017.646
27 vs 16	321253.855	2.214	3.826	0.946		No	-233837.739	876345.448
27 vs 15	157510.595	1.086	3.826	1.000		No	-397580.998	712602.189
27 vs 14	124247.820	0.856	3.826	1.000		No	-430843.774	679339.414
27 vs 13	649379.654	4.476	3.826	0.004	**	Yes	94288.061	1204471.248
27 vs 10	174704.347	1.204	3.826	1.000		No	-380387.246	729795.941
26 vs ZA	784378.723	5.407	3.826	<0.0001	***	Yes	229287.129	1339470.316
26 vs PBS	163199.097	1.125	3.826	1.000		No	-391892.497	718290.690

26 vs 9	62666.224	0.432	3.826	1.000		No	-492425.369	617757.818
26 vs 8	221420.177	1.526	3.826	1.000		No	-333671.417	776511.771
26 vs 7	324644.870	2.238	3.826	0.939		No	-230446.724	879736.464
26 vs 6	709772.299	4.892	3.826	0.001	***	Yes	154680.706	1264863.893
26 vs 5	249783.330	1.722	3.826	0.999		No	-305308.264	804874.924
26 vs 4	108384.409	0.747	3.826	1.000		No	-446707.185	663476.003
26 vs 31	368297.068	2.539	3.826	0.793		No	-186794.525	923388.662
26 vs 30	238725.538	1.645	3.826	0.999		No	-316366.056	793817.131
26 vs 3	542159.946	3.737	3.826	0.067	°	No	-12931.647	1097251.540
26 vs 29	139159.967	0.959	3.826	1.000		No	-415931.627	694251.561
26 vs 28	50828.849	0.350	3.826	1.000		No	-504262.744	605920.443
26 vs 27	30604.450	0.211	3.826	1.000		No	-524487.144	585696.044
26 vs 25	159336.833	1.098	3.826	1.000		No	-395754.761	714428.427
26 vs 24	90054.466	0.621	3.826	1.000		No	-465037.128	645146.060
26 vs 23	51391.033	0.354	3.826	1.000		No	-503700.561	606482.627
26 vs 21	91576.127	0.631	3.826	1.000		No	-463515.466	646667.721
26 vs 2	235142.921	1.621	3.826	1.000		No	-319948.673	790234.514
26 vs 19	546023.515	3.764	3.826	0.061	°	No	-9068.079	1101115.109
26 vs 18	341530.503	2.354	3.826	0.895		No	-213561.091	896622.097
26 vs 17	16525.014	0.114	3.826	1.000		No	-538566.580	571616.608
26 vs 16	351858.305	2.425	3.826	0.860		No	-203233.289	906949.899
26 vs 15	188115.046	1.297	3.826	1.000		No	-366976.548	743206.639
26 vs 14	154852.270	1.067	3.826	1.000		No	-400239.324	709943.864
26 vs 13	679984.105	4.687	3.826	0.002	**	Yes	124892.511	1235075.698
26 vs 10	205308.798	1.415	3.826	1.000		No	-349782.796	760400.391
25 vs ZA	625041.889	4.308	3.826	0.009	**	Yes	69950.296	1180133.483
25 vs PBS	3862.263	0.027	3.826	1.000		No	-551229.330	558953.857
25 vs 8	62083.344	0.428	3.826	1.000		No	-493008.250	617174.938
25 vs 7	165308.037	1.139	3.826	1.000		No	-389783.557	720399.631
25 vs 6	550435.466	3.794	3.826	0.056	°	No	-4656.128	1105527.060
25 vs 5	90446.497	0.623	3.826	1.000		No	-464645.097	645538.090
25 vs 31	208960.235	1.440	3.826	1.000		No	-346131.359	764051.829
25 vs 30	79388.704	0.547	3.826	1.000		No	-475702.889	634480.298
25 vs 3	382823.113	2.639	3.826	0.722		No	-172268.481	937914.707
25 vs 2	75806.087	0.523	3.826	1.000		No	-479285.506	630897.681
25 vs 19	386686.682	2.665	3.826	0.702		No	-168404.912	941778.276
25 vs 18	182193.670	1.256	3.826	1.000		No	-372897.924	737285.263
25 vs 16	192521.472	1.327	3.826	1.000		No	-362570.122	747613.065
25 vs 15	28778.212	0.198	3.826	1.000		No	-526313.381	583869.806
25 vs 13	520647.271	3.589	3.826	0.105		No	-34444.322	1075738.865
25 vs 10	45971.964	0.317	3.826	1.000		No	-509119.629	601063.558
24 vs ZA	694324.257	4.786	3.826	0.001	**	Yes	139232.663	1249415.850
24 vs PBS	73144.631	0.504	3.826	1.000		No	-481946.963	628236.224
24 vs 8	131365.711	0.905	3.826	1.000		No	-423725.883	686457.305

24 vs 7	234590.404	1.617	3.826	1.000		No	-320501.190	789681.998
24 vs 6	619717.833	4.272	3.826	0.010	**	Yes	64626.240	1174809.427
24 vs 5	159728.864	1.101	3.826	1.000		No	-395362.730	714820.458
24 vs 4	18329.943	0.126	3.826	1.000		No	-536761.651	573421.537
24 vs 31	278242.602	1.918	3.826	0.992		No	-276848.991	833334.196
24 vs 30	148671.072	1.025	3.826	1.000		No	-406420.522	703762.665
24 vs 3	452105.480	3.116	3.826	0.345		No	-102986.113	1007197.074
24 vs 29	49105.501	0.338	3.826	1.000		No	-505986.093	604197.095
24 vs 25	69282.367	0.478	3.826	1.000		No	-485809.227	624373.961
24 vs 21	1521.661	0.010	3.826	1.000		No	-553569.932	556613.255
24 vs 2	145088.455	1.000	3.826	1.000		No	-410003.139	700180.048
24 vs 19	455969.049	3.143	3.826	0.326		No	-99122.545	1011060.643
24 vs 18	251476.037	1.733	3.826	0.999		No	-303615.557	806567.631
24 vs 16	261803.839	1.805	3.826	0.997		No	-293287.755	816895.433
24 vs 15	98060.580	0.676	3.826	1.000		No	-457031.014	653152.173
24 vs 14	64797.804	0.447	3.826	1.000		No	-490293.790	619889.398
24 vs 13	589929.639	4.066	3.826	0.022	*	Yes	34838.045	1145021.232
24 vs 10	115254.332	0.794	3.826	1.000		No	-439837.262	670345.925
23 vs ZA	732987.690	5.052	3.826	0.000	***	Yes	177896.096	1288079.283
23 vs PBS	111808.064	0.771	3.826	1.000		No	-443283.530	666899.657
23 vs 9	11275.191	0.078	3.826	1.000		No	-543816.403	566366.785
23 vs 8	170029.144	1.172	3.826	1.000		No	-385062.450	725120.738
23 vs 7	273253.837	1.883	3.826	0.994		No	-281837.757	828345.431
23 vs 6	658381.266	4.538	3.826	0.003	**	Yes	103289.672	1213472.860
23 vs 5	198392.297	1.367	3.826	1.000		No	-356699.297	753483.890
23 vs 4	56993.376	0.393	3.826	1.000		No	-498098.218	612084.970
23 vs 31	316906.035	2.184	3.826	0.954		No	-238185.559	871997.629
23 vs 30	187334.504	1.291	3.826	1.000		No	-367757.089	742426.098
23 vs 3	490768.913	3.383	3.826	0.186		No	-64322.680	1045860.507
23 vs 29	87768.934	0.605	3.826	1.000		No	-467322.660	642860.528
23 vs 25	107945.800	0.744	3.826	1.000		No	-447145.794	663037.394
23 vs 24	38663.433	0.266	3.826	1.000		No	-516428.161	593755.027
23 vs 21	40185.094	0.277	3.826	1.000		No	-514906.499	595276.688
23 vs 2	183751.888	1.267	3.826	1.000		No	-371339.706	738843.481
23 vs 19	494632.482	3.409	3.826	0.173		No	-60459.112	1049724.076
23 vs 18	290139.470	2.000	3.826	0.985		No	-264952.124	845231.063
23 vs 16	300467.272	2.071	3.826	0.977		No	-254624.322	855558.865
23 vs 15	136724.013	0.942	3.826	1.000		No	-418367.581	691815.606
23 vs 14	103461.237	0.713	3.826	1.000		No	-451630.357	658552.831
23 vs 13	628593.072	4.333	3.826	0.008	**	Yes	73501.478	1183684.665
23 vs 10	153917.764	1.061	3.826	1.000		No	-401173.829	709009.358
22 vs ZA	785517.875	5.414	3.826	<0.0001	***	Yes	230426.282	1340609.469
22 vs PBS	164338.249	1.133	3.826	1.000		No	-390753.344	719429.843
22 vs 9	63805.377	0.440	3.826	1.000		No	-491286.217	618896.971

22 vs 8	222559.330	1.534	3.826	1.000		No	-332532.264	777650.923
22 vs 7	325784.023	2.246	3.826	0.937		No	-229307.571	880875.617
22 vs 6	710911.452	4.900	3.826	0.001	***	Yes	155819.858	1266003.046
22 vs 5	250922.482	1.730	3.826	0.999		No	-304169.111	806014.076
22 vs 4	109523.562	0.755	3.826	1.000		No	-445568.032	664615.155
22 vs 31	369436.221	2.546	3.826	0.787		No	-185655.373	924527.815
22 vs 30	239864.690	1.653	3.826	0.999		No	-315226.903	794956.284
22 vs 3	543299.099	3.745	3.826	0.065	°	No	-11792.495	1098390.693
22 vs 29	140299.120	0.967	3.826	1.000		No	-414792.474	695390.714
22 vs 28	51968.002	0.358	3.826	1.000		No	-503123.592	607059.596
22 vs 27	31743.603	0.219	3.826	1.000		No	-523347.991	586835.197
22 vs 26	1139.153	0.008	3.826	1.000		No	-553952.441	556230.747
22 vs 25	160475.986	1.106	3.826	1.000		No	-394615.608	715567.580
22 vs 24	91193.619	0.629	3.826	1.000		No	-463897.975	646285.213
22 vs 23	52530.186	0.362	3.826	1.000		No	-502561.408	607621.780
22 vs 21	92715.280	0.639	3.826	1.000		No	-462376.314	647806.874
22 vs 2	236282.073	1.629	3.826	1.000		No	-318809.520	791373.667
22 vs 19	547162.668	3.771	3.826	0.060	°	No	-7928.926	1102254.262
22 vs 18	342669.655	2.362	3.826	0.892		No	-212421.938	897761.249
22 vs 17	17664.167	0.122	3.826	1.000		No	-537427.427	572755.760
22 vs 16	352997.458	2.433	3.826	0.856		No	-202094.136	908089.051
22 vs 15	189254.198	1.304	3.826	1.000		No	-365837.395	744345.792
22 vs 14	155991.423	1.075	3.826	1.000		No	-399100.171	711083.016
22 vs 13	681123.257	4.695	3.826	0.002	**	Yes	126031.664	1236214.851
22 vs 10	206447.950	1.423	3.826	1.000		No	-348643.643	761539.544
21 vs ZA	692802.595	4.775	3.826	0.001	**	Yes	137711.001	1247894.189
21 vs PBS	71622.969	0.494	3.826	1.000		No	-483468.625	626714.563
21 vs 8	129844.050	0.895	3.826	1.000		No	-425247.544	684935.643
21 vs 7	233068.743	1.606	3.826	1.000		No	-322022.851	788160.337
21 vs 6	618196.172	4.261	3.826	0.010	*	Yes	63104.578	1173287.766
21 vs 5	158207.202	1.090	3.826	1.000		No	-396884.391	713298.796
21 vs 4	16808.281	0.116	3.826	1.000		No	-538283.312	571899.875
21 vs 31	276720.941	1.907	3.826	0.993		No	-278370.653	831812.535
21 vs 30	147149.410	1.014	3.826	1.000		No	-407942.184	702241.004
21 vs 3	450583.819	3.106	3.826	0.352		No	-104507.775	1005675.413
21 vs 29	47583.840	0.328	3.826	1.000		No	-507507.754	602675.434
21 vs 25	67760.706	0.467	3.826	1.000		No	-487330.888	622852.300
21 vs 2	143566.793	0.990	3.826	1.000		No	-411524.801	698658.387
21 vs 19	454447.388	3.132	3.826	0.333		No	-100644.206	1009538.981
21 vs 18	249954.375	1.723	3.826	0.999		No	-305137.218	805045.969
21 vs 16	260282.177	1.794	3.826	0.997		No	-294809.416	815373.771
21 vs 15	96538.918	0.665	3.826	1.000		No	-458552.676	651630.512
21 vs 14	63276.143	0.436	3.826	1.000		No	-491815.451	618367.736
21 vs 13	588407.977	4.056	3.826	0.022	*	Yes	33316.383	1143499.571

21 vs 10	113732.670	0.784	3.826	1.000		No	-441358.924	668824.264
20 vs ZA	1320757.614	9.104	3.826	<0.0001	***	Yes	765666.020	1875849.208
20 vs PBS	699577.988	4.822	3.826	0.001	***	Yes	144486.394	1254669.582
20 vs DMSO 3%	523796.643	3.610	3.826	0.050	*	Yes	-31294.950	1078888.237
20 vs 9	599045.116	4.129	3.826	0.017	*	Yes	43953.522	1154136.709
20 vs 8	757799.068	5.223	3.826	0.000	***	Yes	202707.474	1312890.662
20 vs 7	861023.762	5.935	3.826	<0.0001	***	Yes	305932.168	1416115.355
20 vs 6	1246151.191	8.589	3.826	<0.0001	***	Yes	691059.597	1801242.784
20 vs 5	786162.221	5.419	3.826	<0.0001	***	Yes	231070.627	1341253.815
20 vs 4	644763.300	4.444	3.826	0.005	**	Yes	89671.706	1199854.894
20 vs 31	904675.960	6.236	3.826	<0.0001	***	Yes	349584.366	1459767.553
20 vs 30	775104.429	5.343	3.826	<0.0001	***	Yes	220012.835	1330196.023
20 vs 3	1078538.838	7.434	3.826	<0.0001	***	Yes	523447.244	1633630.431
20 vs 29	675538.859	4.656	3.826	0.002	**	Yes	120447.265	1230630.452
20 vs 28	587207.741	4.047	3.826	0.023	*	Yes	32116.147	1142299.335
20 vs 27	566983.342	3.908	3.826	0.038	*	Yes	11891.748	1122074.935
20 vs 26	536378.891	3.697	3.826	0.076		No	-18712.702	1091470.485
20 vs 25	695715.724	4.795	3.826	0.001	**	Yes	140624.131	1250807.318
20 vs 24	626433.357	4.318	3.826	0.008	**	Yes	71341.764	1181524.951
20 vs 23	587769.924	4.051	3.826	0.023	*	Yes	32678.331	1142861.518
20 vs 22	535239.739	3.689	3.826	0.078		No	-19851.855	1090331.332
20 vs 21	627955.019	4.328	3.826	0.008	**	Yes	72863.425	1183046.613
20 vs 2	771521.812	5.318	3.826	<0.0001	***	Yes	216430.218	1326613.406
20 vs 19	1082402.406	7.461	3.826	<0.0001	***	Yes	527310.813	1637494.000
20 vs 18	877909.394	6.051	3.826	<0.0001	***	Yes	322817.800	1433000.988
20 vs 17	552903.905	3.811	3.826	0.053	°	No	-2187.689	1107995.499
20 vs 16	888237.196	6.122	3.826	<0.0001	***	Yes	333145.602	1443328.790
20 vs 15	724493.937	4.994	3.826	0.000	***	Yes	169402.343	1279585.531
20 vs 14	691231.161	4.764	3.826	0.001	**	Yes	136139.567	1246322.755
20 vs 13	1216362.996	8.384	3.826	<0.0001	***	Yes	661271.402	1771454.590
20 vs 12	519745.907	3.582	3.826	0.107		No	-35345.686	1074837.501
20 vs 11	348959.489	2.405	3.826	0.871		No	-206132.105	904051.083
20 vs 10	741687.689	5.112	3.826	0.000	***	Yes	186596.095	1296779.283
2 vs ZA	549235.802	3.786	3.826	0.057	°	No	-5855.792	1104327.396
2 vs 7	89501.950	0.617	3.826	1.000		No	-465589.644	644593.543
2 vs 6	474629.379	3.271	3.826	0.244		No	-80462.215	1029720.972
2 vs 5	14640.409	0.101	3.826	1.000		No	-540451.185	569732.003
2 vs 31	133154.148	0.918	3.826	1.000		No	-421937.446	688245.741
2 vs 30	3582.617	0.025	3.826	1.000		No	-551508.977	558674.211
2 vs 3	307017.026	2.116	3.826	0.969		No	-248074.568	862108.620
2 vs 19	310880.594	2.143	3.826	0.964		No	-244210.999	865972.188
2 vs 18	106387.582	0.733	3.826	1.000		No	-448704.012	661479.176
2 vs 16	116715.384	0.804	3.826	1.000		No	-438376.210	671806.978
2 vs 13	444841.184	3.066	3.826	0.381		No	-110250.410	999932.778

19 vs ZA	238355.208	1.643	3.826	0.999		No	-316736.386	793446.801
19 vs 6	163748.784	1.129	3.826	1.000		No	-391342.810	718840.378
19 vs 13	133960.590	0.923	3.826	1.000		No	-421131.004	689052.183
18 vs ZA	442848.220	3.052	3.826	0.391		No	-112243.374	997939.814
18 vs 6	368241.797	2.538	3.826	0.793		No	-186849.797	923333.390
18 vs 31	26766.566	0.184	3.826	1.000		No	-528325.028	581858.159
18 vs 3	200629.444	1.383	3.826	1.000		No	-354462.150	755721.037
18 vs 19	204493.012	1.410	3.826	1.000		No	-350598.581	759584.606
18 vs 16	10327.802	0.071	3.826	1.000		No	-544763.792	565419.396
18 vs 13	338453.602	2.333	3.826	0.904		No	-216637.992	893545.196
17 vs ZA	767853.709	5.293	3.826	0.000	***	Yes	212762.115	1322945.302
17 vs PBS	146674.083	1.011	3.826	1.000		No	-408417.511	701765.676
17 vs 9	46141.210	0.318	3.826	1.000		No	-508950.383	601232.804
17 vs 8	204895.163	1.412	3.826	1.000		No	-350196.431	759986.757
17 vs 7	308119.856	2.124	3.826	0.967		No	-246971.738	863211.450
17 vs 6	693247.285	4.778	3.826	0.001	**	Yes	138155.692	1248338.879
17 vs 5	233258.316	1.608	3.826	1.000		No	-321833.278	788349.910
17 vs 4	91859.395	0.633	3.826	1.000		No	-463232.199	646950.989
17 vs 31	351772.054	2.425	3.826	0.861		No	-203319.539	906863.648
17 vs 30	222200.524	1.532	3.826	1.000		No	-332891.070	777292.117
17 vs 3	525634.932	3.623	3.826	0.095	°	No	-29456.661	1080726.526
17 vs 29	122634.953	0.845	3.826	1.000		No	-432456.640	677726.547
17 vs 28	34303.835	0.236	3.826	1.000		No	-520787.758	589395.429
17 vs 27	14079.436	0.097	3.826	1.000		No	-541012.158	569171.030
17 vs 25	142811.819	0.984	3.826	1.000		No	-412279.775	697903.413
17 vs 24	73529.452	0.507	3.826	1.000		No	-481562.142	628621.046
17 vs 23	34866.019	0.240	3.826	1.000		No	-520225.575	589957.613
17 vs 21	75051.113	0.517	3.826	1.000		No	-480040.480	630142.707
17 vs 2	218617.907	1.507	3.826	1.000		No	-336473.687	773709.500
17 vs 19	529498.501	3.650	3.826	0.088	°	No	-25593.093	1084590.095
17 vs 18	325005.489	2.240	3.826	0.938		No	-230086.105	880097.083
17 vs 16	335333.291	2.311	3.826	0.913		No	-219758.303	890424.885
17 vs 15	171590.032	1.183	3.826	1.000		No	-383501.562	726681.625
17 vs 14	138327.256	0.953	3.826	1.000		No	-416764.338	693418.850
17 vs 13	663459.091	4.573	3.826	0.003	**	Yes	108367.497	1218550.684
17 vs 10	188783.784	1.301	3.826	1.000		No	-366307.810	743875.377
16 vs ZA	432520.418	2.981	3.826	0.446		No	-122571.176	987612.012
16 vs 6	357913.995	2.467	3.826	0.837		No	-197177.599	913005.588
16 vs 31	16438.764	0.113	3.826	1.000		No	-538652.830	571530.357
16 vs 3	190301.642	1.312	3.826	1.000		No	-364789.952	745393.235
16 vs 19	194165.210	1.338	3.826	1.000		No	-360926.383	749256.804
16 vs 13	328125.800	2.262	3.826	0.931		No	-226965.794	883217.394
15 vs ZA	596263.677	4.110	3.826	0.018	*	Yes	41172.083	1151355.271
15 vs 8	33305.131	0.230	3.826	1.000		No	-521786.462	588396.725

15 vs 7	136529.825	0.941	3.826	1.000		No	-418561.769	691621.418
15 vs 6	521657.254	3.596	3.826	0.103		No	-33434.340	1076748.847
15 vs 5	61668.284	0.425	3.826	1.000		No	-493423.310	616759.878
15 vs 31	180182.023	1.242	3.826	1.000		No	-374909.571	735273.616
15 vs 30	50610.492	0.349	3.826	1.000		No	-504481.102	605702.086
15 vs 3	354044.901	2.440	3.826	0.852		No	-201046.693	909136.495
15 vs 2	47027.875	0.324	3.826	1.000		No	-508063.719	602119.469
15 vs 19	357908.469	2.467	3.826	0.837		No	-197183.124	913000.063
15 vs 18	153415.457	1.057	3.826	1.000		No	-401676.137	708507.051
15 vs 16	163743.259	1.129	3.826	1.000		No	-391348.335	718834.853
15 vs 13	491869.059	3.390	3.826	0.182		No	-63222.535	1046960.653
15 vs 10	17193.752	0.119	3.826	1.000		No	-537897.842	572285.346
14 vs ZA	629526.453	4.339	3.826	0.008	**	Yes	74434.859	1184618.046
14 vs PBS	8346.827	0.058	3.826	1.000		No	-546744.767	563438.420
14 vs 8	66567.907	0.459	3.826	1.000		No	-488523.687	621659.501
14 vs 7	169792.600	1.170	3.826	1.000		No	-385298.994	724884.194
14 vs 6	554920.029	3.825	3.826	0.050	*	Yes	-171.564	1110011.623
14 vs 5	94931.060	0.654	3.826	1.000		No	-460160.534	650022.654
14 vs 31	213444.798	1.471	3.826	1.000		No	-341646.795	768536.392
14 vs 30	83873.268	0.578	3.826	1.000		No	-471218.326	638964.861
14 vs 3	387307.676	2.670	3.826	0.699		No	-167783.917	942399.270
14 vs 25	4484.563	0.031	3.826	1.000		No	-550607.031	559576.157
14 vs 2	80290.651	0.553	3.826	1.000		No	-474800.943	635382.244
14 vs 19	391171.245	2.696	3.826	0.678		No	-163920.349	946262.839
14 vs 18	186678.233	1.287	3.826	1.000		No	-368413.361	741769.827
14 vs 16	197006.035	1.358	3.826	1.000		No	-358085.559	752097.629
14 vs 15	33262.776	0.229	3.826	1.000		No	-521828.818	588354.369
14 vs 13	525131.835	3.620	3.826	0.096	°	No	-29959.759	1080223.428
14 vs 10	50456.528	0.348	3.826	1.000		No	-504635.066	605548.121
13 vs ZA	104394.618	0.720	3.826	1.000		No	-450696.976	659486.212
13 vs 6	29788.195	0.205	3.826	1.000		No	-525303.399	584879.788
12 vs ZA	801011.707	5.521	3.826	<0.0001	***	Yes	245920.113	1356103.300
12 vs PBS	179832.081	1.240	3.826	1.000		No	-375259.513	734923.674
12 vs DMSO 3%	4050.736	0.028	3.826	1.000		No	-551040.858	559142.330
12 vs 9	79299.208	0.547	3.826	1.000		No	-475792.385	634390.802
12 vs 8	238053.161	1.641	3.826	0.999		No	-317038.433	793144.755
12 vs 7	341277.854	2.352	3.826	0.896		No	-213813.740	896369.448
12 vs 6	726405.283	5.007	3.826	0.000	***	Yes	171313.690	1281496.877
12 vs 5	266416.314	1.836	3.826	0.996		No	-288675.280	821507.908
12 vs 4	125017.393	0.862	3.826	1.000		No	-430074.201	680108.987
12 vs 31	384930.052	2.653	3.826	0.711		No	-170161.541	940021.646
12 vs 30	255358.522	1.760	3.826	0.998		No	-299733.072	810450.115
12 vs 3	558792.930	3.852	3.826	0.046	*	Yes	3701.337	1113884.524
12 vs 29	155792.951	1.074	3.826	1.000		No	-399298.643	710884.545

12 vs 28	67461.833	0.465	3.826	1.000		No	-487629.760	622553.427
12 vs 27	47237.434	0.326	3.826	1.000		No	-507854.160	602329.028
12 vs 26	16632.984	0.115	3.826	1.000		No	-538458.610	571724.578
12 vs 25	175969.817	1.213	3.826	1.000		No	-379121.777	731061.411
12 vs 24	106687.450	0.735	3.826	1.000		No	-448404.144	661779.044
12 vs 23	68024.017	0.469	3.826	1.000		No	-487067.577	623115.611
12 vs 22	15493.831	0.107	3.826	1.000		No	-539597.763	570585.425
12 vs 21	108209.111	0.746	3.826	1.000		No	-446882.482	663300.705
12 vs 2	251775.905	1.735	3.826	0.998		No	-303315.689	806867.498
12 vs 19	562656.499	3.878	3.826	0.042	*	Yes	7564.905	1117748.093
12 vs 18	358163.487	2.469	3.826	0.836		No	-196928.107	913255.081
12 vs 17	33157.998	0.229	3.826	1.000		No	-521933.596	588249.592
12 vs 16	368491.289	2.540	3.826	0.792		No	-186600.305	923582.883
12 vs 15	204748.030	1.411	3.826	1.000		No	-350343.564	759839.623
12 vs 14	171485.254	1.182	3.826	1.000		No	-383606.340	726576.848
12 vs 13	696617.089	4.802	3.826	0.001	**	Yes	141525.495	1251708.682
12 vs 10	221941.782	1.530	3.826	1.000		No	-333149.812	777033.375
11 vs ZA	971798.125	6.698	3.826	<0.0001	***	Yes	416706.531	1526889.719
11 vs PBS	350618.499	2.417	3.826	0.865		No	-204473.095	905710.093
11 vs DMSO 3%	174837.154	1.205	3.826	1.000		No	-380254.439	729928.748
11 vs 9	250085.627	1.724	3.826	0.999		No	-305005.967	805177.220
11 vs 8	408839.579	2.818	3.826	0.579		No	-146252.015	963931.173
11 vs 7	512064.272	3.530	3.826	0.125		No	-43027.321	1067155.866
11 vs 6	897191.702	6.184	3.826	<0.0001	***	Yes	342100.108	1452283.295
11 vs 5	437202.732	3.014	3.826	0.421		No	-117888.862	992294.326
11 vs 4	295803.811	2.039	3.826	0.981		No	-259287.783	850895.405
11 vs 31	555716.471	3.830	3.826	0.049	*	Yes	624.877	1110808.064
11 vs 30	426144.940	2.937	3.826	0.482		No	-128946.654	981236.534
11 vs 3	729579.349	5.029	3.826	0.000	***	Yes	174487.755	1284670.942
11 vs 29	326579.370	2.251	3.826	0.935		No	-228512.224	881670.963
11 vs 28	238248.252	1.642	3.826	0.999		No	-316843.342	793339.845
11 vs 27	218023.852	1.503	3.826	1.000		No	-337067.741	773115.446
11 vs 26	187419.402	1.292	3.826	1.000		No	-367672.192	742510.996
11 vs 25	346756.235	2.390	3.826	0.878		No	-208335.358	901847.829
11 vs 24	277473.868	1.913	3.826	0.992		No	-277617.726	832565.462
11 vs 23	238810.435	1.646	3.826	0.999		No	-316281.158	793902.029
11 vs 22	186280.249	1.284	3.826	1.000		No	-368811.344	741371.843
11 vs 21	278995.530	1.923	3.826	0.992		No	-276096.064	834087.123
11 vs 2	422562.323	2.913	3.826	0.502		No	-132529.271	977653.917
11 vs 19	733442.917	5.055	3.826	0.000	***	Yes	178351.324	1288534.511
11 vs 18	528949.905	3.646	3.826	0.089	°	No	-26141.689	1084041.499
11 vs 17	203944.416	1.406	3.826	1.000		No	-351147.178	759036.010
11 vs 16	539277.707	3.717	3.826	0.071	°	No	-15813.887	1094369.301
11 vs 15	375534.448	2.588	3.826	0.759		No	-179557.146	930626.042

11 vs 14	342271.672	2.359	3.826	0.893		No	-212819.922	897363.266
11 vs 13	867403.507	5.979	3.826	<0.0001	***	Yes	312311.913	1422495.101
11 vs 12	170786.418	1.177	3.826	1.000		No	-384305.176	725878.012
11 vs 10	392728.200	2.707	3.826	0.669		No	-162363.394	947819.794
10 vs ZA	579069.925	3.991	3.826	0.028	*	Yes	23978.331	1134161.519
10 vs 8	16111.379	0.111	3.826	1.000		No	-538980.214	571202.973
10 vs 7	119336.073	0.823	3.826	1.000		No	-435755.521	674427.666
10 vs 6	504463.502	3.477	3.826	0.144		No	-50628.092	1059555.096
10 vs 5	44474.532	0.307	3.826	1.000		No	-510617.062	599566.126
10 vs 31	162988.271	1.123	3.826	1.000		No	-392103.323	718079.865
10 vs 30	33416.740	0.230	3.826	1.000		No	-521674.854	588508.334
10 vs 3	336851.149	2.322	3.826	0.909		No	-218240.445	891942.743
10 vs 2	29834.123	0.206	3.826	1.000		No	-525257.471	584925.717
10 vs 19	340714.718	2.348	3.826	0.898		No	-214376.876	895806.311
10 vs 18	136221.705	0.939	3.826	1.000		No	-418869.889	691313.299
10 vs 16	146549.507	1.010	3.826	1.000		No	-408542.087	701641.101
10 vs 13	474675.307	3.272	3.826	0.244		No	-80416.287	1029766.901

Table S2c

Category	Groups								
11	A	B							
12	A	B	C						
DMSO 3%	A	B	C	D					
22	A	B	C	D	E				
26	A	B	C	D	E				
17	A	B	C	D	E				
27		B	C	D	E				
28		B	C	D	E				
23		B	C	D	E				
9		B	C	D	E				
24		B	C	D	E				
21		B	C	D	E				
4		B	C	D	E				
29		B	C	D	E	F			
14		B	C	D	E	F	G		
25		B	C	D	E	F	G		
PBS		B	C	D	E	F	G		
15		B	C	D	E	F	G		
10		B	C	D	E	F	G		
8		B	C	D	E	F	G		
2		B	C	D	E	F	G	H	
30		B	C	D	E	F	G	H	
5		B	C	D	E	F	G	H	
7		B	C	D	E	F	G	H	
18		B	C	D	E	F	G	H	
16		B	C	D	E	F	G	H	
31			C	D	E	F	G	H	
3					E	F	G	H	
19					E	F	G	H	
13						F	G	H	
6							G	H	
ZA								H	
20									I

Table S3. Bray-Curtis proximity matrix obtained from averages of ZA-analogues adhesion data. The more similar is the fungal adhesion, the closer is the distance in the score matrix.

	1 (ZA)	6	13	19	3	31	18	7	5	30	2	8	10	15	PBS	25	14	29	4	21	24	9	23	28	27	17	26	22	DMSO 3%	12	16	11	20
1 (ZA)	0.000	0.094	0.126	0.248	0.251	0.366	0.380	0.389	0.425	0.430	0.432	0.438	0.445	0.452	0.462	0.464	0.466	0.472	0.484	0.490	0.490	0.500	0.504	0.504	0.511	0.515	0.521	0.521	0.525	0.526	0.542	0.574	0.647
6	0.094	0.000	0.033	0.158	0.161	0.282	0.297	0.307	0.346	0.351	0.353	0.359	0.367	0.374	0.385	0.387	0.389	0.396	0.408	0.415	0.416	0.426	0.430	0.431	0.438	0.443	0.449	0.449	0.453	0.455	0.473	0.507	0.589
13	0.126	0.033	0.000	0.126	0.129	0.251	0.267	0.276	0.316	0.322	0.323	0.330	0.338	0.346	0.357	0.359	0.361	0.367	0.380	0.387	0.388	0.399	0.403	0.403	0.411	0.416	0.422	0.423	0.427	0.428	0.447	0.482	0.566
19	0.248	0.158	0.126	0.000	0.003	0.129	0.146	0.156	0.198	0.204	0.206	0.213	0.221	0.230	0.242	0.244	0.246	0.253	0.267	0.275	0.276	0.287	0.292	0.292	0.301	0.306	0.313	0.313	0.318	0.319	0.340	0.380	0.474
3	0.251	0.161	0.129	0.003	0.000	0.126	0.143	0.153	0.195	0.201	0.203	0.210	0.218	0.227	0.239	0.241	0.243	0.250	0.264	0.272	0.273	0.284	0.289	0.289	0.298	0.303	0.310	0.310	0.315	0.317	0.337	0.377	0.472
31	0.366	0.282	0.251	0.129	0.126	0.000	0.017	0.027	0.071	0.077	0.079	0.086	0.095	0.104	0.117	0.119	0.121	0.128	0.143	0.151	0.152	0.164	0.169	0.170	0.178	0.185	0.192	0.192	0.197	0.198	0.221	0.263	0.368
18	0.380	0.297	0.267	0.146	0.143	0.017	0.000	0.010	0.054	0.060	0.062	0.070	0.078	0.087	0.100	0.102	0.104	0.112	0.127	0.135	0.135	0.148	0.153	0.153	0.162	0.168	0.175	0.176	0.180	0.182	0.205	0.248	0.353
7	0.389	0.307	0.276	0.156	0.153	0.027	0.010	0.000	0.044	0.050	0.052	0.059	0.068	0.077	0.090	0.091	0.094	0.102	0.116	0.124	0.125	0.138	0.143	0.143	0.152	0.158	0.165	0.166	0.170	0.172	0.195	0.238	0.344
5	0.425	0.346	0.316	0.198	0.195	0.071	0.054	0.044	0.000	0.006	0.008	0.016	0.024	0.033	0.046	0.048	0.050	0.058	0.073	0.081	0.082	0.095	0.100	0.100	0.109	0.115	0.122	0.123	0.128	0.129	0.152	0.196	0.305
30	0.430	0.351	0.322	0.204	0.201	0.077	0.060	0.050	0.006	0.000	0.002	0.009	0.018	0.027	0.040	0.042	0.044	0.052	0.067	0.075	0.076	0.088	0.094	0.094	0.103	0.109	0.116	0.117	0.122	0.123	0.146	0.190	0.299
2	0.432	0.353	0.323	0.206	0.203	0.079	0.062	0.052	0.008	0.002	0.000	0.007	0.016	0.025	0.038	0.040	0.042	0.050	0.065	0.073	0.074	0.087	0.092	0.092	0.101	0.107	0.114	0.115	0.120	0.121	0.144	0.188	0.298
8	0.438	0.359	0.330	0.213	0.210	0.086	0.070	0.059	0.016	0.009	0.007	0.000	0.009	0.018	0.031	0.033	0.035	0.043	0.058	0.066	0.066	0.079	0.084	0.085	0.094	0.100	0.107	0.107	0.112	0.114	0.137	0.181	0.291
10	0.445	0.367	0.338	0.221	0.218	0.095	0.078	0.068	0.024	0.018	0.016	0.009	0.000	0.009	0.022	0.024	0.026	0.034	0.049	0.057	0.058	0.071	0.076	0.076	0.085	0.091	0.098	0.099	0.104	0.106	0.128	0.173	0.283
15	0.452	0.374	0.346	0.230	0.227	0.104	0.087	0.077	0.033	0.027	0.025	0.018	0.009	0.000	0.013	0.015	0.017	0.025	0.040	0.048	0.049	0.061	0.067	0.067	0.076	0.082	0.089	0.090	0.095	0.097	0.120	0.164	0.275
PBS	0.462	0.385	0.357	0.242	0.239	0.117	0.100	0.090	0.046	0.040	0.038	0.031	0.022	0.013	0.000	0.002	0.004	0.012	0.027	0.035	0.036	0.049	0.054	0.054	0.063	0.069	0.077	0.077	0.082	0.084	0.107	0.151	0.263
25	0.464	0.387	0.359	0.244	0.241	0.119	0.102	0.091	0.048	0.042	0.040	0.033	0.024	0.015	0.002	0.000	0.002	0.010	0.025	0.033	0.034	0.047	0.052	0.052	0.061	0.068	0.075	0.075	0.080	0.082	0.105	0.150	0.261
14	0.466	0.389	0.361	0.246	0.243	0.121	0.104	0.094	0.050	0.044	0.042	0.035	0.026	0.017	0.004	0.002	0.000	0.008	0.023	0.031	0.032	0.044	0.050	0.050	0.059	0.065	0.072	0.073	0.078	0.080	0.103	0.147	0.259
29	0.472	0.396	0.367	0.253	0.250	0.128	0.112	0.102	0.058	0.052	0.050	0.043	0.034	0.025	0.012	0.010	0.008	0.000	0.015	0.023	0.024	0.037	0.042	0.042	0.051	0.057	0.065	0.065	0.070	0.072	0.095	0.140	0.251
4	0.484	0.408	0.380	0.267	0.264	0.143	0.127	0.116	0.073	0.067	0.065	0.058	0.049	0.040	0.027	0.025	0.023	0.015	0.000	0.008	0.009	0.022	0.027	0.027	0.036	0.042	0.050	0.050	0.055	0.057	0.080	0.125	0.237
21	0.490	0.415	0.387	0.275	0.272	0.151	0.135	0.124	0.081	0.075	0.073	0.066	0.057	0.048	0.035	0.033	0.031	0.023	0.008	0.000	0.001	0.014	0.019	0.019	0.028	0.034	0.042	0.042	0.047	0.049	0.072	0.117	0.230
24	0.490	0.416	0.388	0.276	0.273	0.152	0.135	0.125	0.082	0.076	0.074	0.066	0.058	0.049	0.036	0.034	0.032	0.024	0.009	0.001	0.000	0.013	0.018	0.018	0.027	0.034	0.041	0.041	0.046	0.048	0.071	0.116	0.229
9	0.500	0.426	0.399	0.287	0.284	0.164	0.148	0.138	0.095	0.088	0.087	0.079	0.071	0.061	0.049	0.047	0.044	0.037	0.022	0.014	0.013	0.000	0.005	0.005	0.015	0.021	0.028	0.029	0.034	0.035	0.059	0.104	0.217
23	0.504	0.430	0.403	0.292	0.289	0.169	0.153	0.143	0.100	0.094	0.092	0.084	0.076	0.067	0.054	0.052	0.050	0.042	0.027	0.019	0.018	0.005	0.000	0.000	0.009	0.016	0.023	0.023	0.028	0.030	0.053	0.098	0.212
28	0.504	0.431	0.403	0.292	0.289	0.170	0.153	0.143	0.100	0.094	0.092	0.085	0.076	0.067	0.054	0.052	0.050	0.042	0.027	0.019	0.018	0.005	0.000	0.000	0.009	0.015	0.023	0.023	0.028	0.030	0.053	0.098	0.211
27	0.511	0.438	0.411	0.301	0.298	0.178	0.162	0.152	0.109	0.103	0.101	0.094	0.085	0.076	0.063	0.061	0.059	0.051	0.036	0.028	0.027	0.015	0.009	0.009	0.000	0.006	0.014	0.014	0.019	0.021	0.044	0.089	0.203
17	0.515	0.443	0.416	0.306	0.303	0.185	0.168	0.158	0.115	0.109	0.107	0.100	0.091	0.082	0.069	0.068	0.065	0.057	0.042	0.034	0.034	0.021	0.016	0.015	0.006	0.000	0.007	0.008	0.013	0.014	0.038	0.083	0.197
26	0.521	0.449	0.422	0.313	0.310	0.192	0.175	0.165	0.122	0.116	0.114	0.107	0.098	0.089	0.077	0.075	0.072	0.065	0.050	0.042	0.041	0.028	0.023	0.023	0.014	0.007	0.000	0.000	0.005	0.007	0.030	0.076	0.190
22	0.521	0.449	0.423	0.313	0.310	0.192	0.176	0.166	0.123	0.117	0.115	0.107	0.099	0.090	0.077	0.075	0.073	0.065	0.050	0.042	0.041	0.029	0.023	0.023	0.014	0.008	0.000	0.000	0.005	0.007	0.030	0.075	0.189
DMSO 3%	0.525	0.453	0.427	0.318	0.315	0.197	0.180	0.170	0.128	0.122	0.120	0.112	0.104	0.095	0.082	0.080	0.078	0.070	0.055	0.047	0.046	0.034	0.028	0.028	0.019	0.013	0.005	0.005	0.000	0.002	0.025	0.070	0.184
12	0.526	0.455	0.428	0.319	0.317	0.198	0.182	0.172	0.129	0.123	0.121	0.114	0.106	0.097	0.084	0.082	0.080	0.072	0.057	0.049	0.048	0.035	0.030	0.030	0.021	0.014	0.007	0.007	0.002	0.000	0.023	0.068	0.183
16	0.542	0.473	0.447	0.340	0.337	0.221	0.205	0.195	0.152	0.146	0.144	0.137	0.128	0.120	0.107	0.105	0.103	0.095	0.080	0.072	0.071	0.059	0.053	0.053	0.044	0.038	0.030	0.030	0.025	0.023	0.000	0.045	0.160
11	0.574	0.507	0.482	0.380	0.377	0.263	0.248	0.238	0.196	0.190	0.188	0.181	0.173	0.164	0.151	0.150	0.147	0.140	0.125	0.117	0.116	0.104	0.098	0.098	0.089	0.083	0.076	0.075	0.070	0.068	0.045	0.000	0.116
20	0.647	0.589	0.566	0.474	0.472	0.368	0.353	0.344	0.305	0.299	0.298	0.291	0.283	0.275	0.263	0.261	0.259	0.251	0.237	0.230	0.229	0.217	0.212	0.211	0.203	0.197	0.190	0.189	0.184	0.183	0.160	0.116	0.000

Table S4. MANOVA analysis obtained from averages of ZA-analogues adhesion data. Variables and categories considered in the analysis are reported.

Table S4a. Presence of double bond * *Cis/Trans* configuration

Variable	Categories
E/Z Configuration	-
	E
	Z
Double bond presence	no
	yes

**3 vs 4 vs 5**	Double bond presence	E/Z	Double bond presence*E/Z
Lambda	0.000	0.831	0.000
F Observed values	0.000	5.895	0.000
DF1	0	2	0
DF2	0	58	0
F Critical value	0.000	3.156	0.000
p-value	**<0.0001**	**0.005**	**<0.0001**

H0: The variable or the interaction of the corresponding column has no significant effect on the dependent variables.

Ha: The variable or the interaction of the corresponding column has a significant effect on the dependent variables.

Double bond presence: As the computed p-value is lower than the significance level alpha=0.05, one should reject the null hypothesis H0, and accept the alternative hypothesis Ha.

The risk to reject the null hypothesis H0 while it is true is lower than 0.01%.

E/Z: As the computed p-value is lower than the significance level alpha=0.05, one should reject the null hypothesis H0, and accept the alternative hypothesis Ha.

The risk to reject the null hypothesis H0 while it is true is lower than 0.47%.

Table S4b. Presence of double bond * *Cis/Trans* configuration

Variable	Categories
E/Z Configuration	-
	E
	Z
Double bond presence	no
	yes

**13 vs 14 vs 15**	Double bond presence	E/Z	Double bond presence*E/Z
Lambda	0.000	0.744	0.000
F Observed values	0.000	6.380	0.000
DF1	0	2	0
DF2	0	37	0
F Critical value	0.000	3.252	0.000
p-value	**<0.0001**	**0.004**	**<0.0001**

H0: The variable or the interaction of the corresponding column has no significant effect on the dependent variables.

Ha: The variable or the interaction of the corresponding column has a significant effect on the dependent variables.

Double bond presence: As the computed p-value is lower than the significance level alpha=0.05, one should reject the null hypothesis H0, and accept the alternative hypothesis Ha.

The risk to reject the null hypothesis H0 while it is true is lower than 0.01%.
E/Z: As the computed p-value is lower than the significance level alpha=0.05, one should reject the null hypothesis H0. and accept the alternative hypothesis Ha.

The risk to reject the null hypothesis H0 while it is true is lower than 0.42%.

Table S4c. *Cis/Trans* configuration

Variable	Categories
Q2	E
	Z

**ZA vs 2**	E/Z
Lambda	0.500
F Observed values	35.996
DF1	1
DF2	36
F Critical value	4.113
p-value	**<0.0001**

H0: The variable or the interaction of the corresponding column has no significant effect on the dependent variables.

Ha: The variable or the interaction of the corresponding column has a significant effect on the dependent variables.
Q2: As the computed p-value is lower than the significance level alpha=0.05, one should reject the null hypothesis H0. and accept the alternative hypothesis Ha.

The risk to reject the null hypothesis H0 while it is true is lower than 0.01%.

Table S4d. Side chain function*COOH side chain presence

Variable	Categories
Side chain function	alcool
	aldheyde
	carboxylic
	ethyl ester
	methyl ester
COOH side chain presence	no
	yes

**3 vs 6 vs 7 vs 10 vs 12**	Side chain function	COOH side chain presence	Side chain function*COOH side chain presence
Lambda	0.419	0.000	0.000
F Observed values	5.539	0.000	0.000
DF1	4	0	0
DF2	16	0	0
F Critical value	3.007	0.000	0.000
p-value	**0.005**	**<0.0001**	**<0.0001**

H0: The variable or the interaction of the corresponding column has no significant effect on the dependent variables.

Ha: The variable or the interaction of the corresponding column has a significant effect on the dependent variables.
Chain function: As the computed p-value is lower than the significance level alpha=0.05, one should reject the null hypothesis H0, and accept the alternative hypothesis Ha.

The risk to reject the null hypothesis H0 while it is true is lower than 0.50%.

COOH side chian: As the computed p-value is lower than the significance level alpha=0.05, one should reject the null hypothesis H0 and accept the alternative hypothesis Ha.

The risk to reject the null hypothesis H0 while it is true is lower than 0.01%.

Table S4e. Position of the substituent*Presence of the substituent

Variable	Categories
Position of the substituent	2-OH
	3-OH
	4-OH
	H
Presence of the substituent	no
	yes

**3 vs 13 vs 17 vs 18**	Position of the substituent	Presence of the substituent	Position of the substituent*Presence of the substituent
Lambda	0.246	0.000	0.000
F Observed values	15.329	0.000	0.000
DF1	3	0	0
DF2	15	0	0
F Critical value	3.287	0.000	0.000
p-value	**<0.0001**	**<0.0001**	**<0.0001**

H0: The variable or the interaction of the corresponding column has no significant effect on the dependent variables.

Ha: The variable or the interaction of the corresponding column has a significant effect on the dependent variables.

Position of the substituent: As the computed p-value is lower than the significance level alpha=0.05, one should reject the null hypothesis H0, and accept the alternative hypothesis Ha.

The risk to reject the null hypothesis H0 while it is true is lower than 0.01%.

Presence of the substituent: As the computed p-value is lower than the significance level alpha=0.05, one should reject the null hypothesis H0, and accept the alternative hypothesis Ha.

The risk to reject the null hypothesis H0 while it is true is lower than 0.01%.

Table S4f. Position of the substituent*Presence of the substituent

Variable	Categories
Position of the substituent	3-Cl
	4-Cl
	H
Presence of the substituent	no
	yes

3 vs 22 vs 23	Position of the substituent	Presence of the substituent	Position of the substituent*Presence of the substituent
Lambda	0.301	0.000	0.000
F Observed values	13.952	0.000	0.000
DF1	2	0	0
DF2	12	0	0
F Critical value	3.885	0.000	0.000
p-value	**0.001**	**<0.0001**	**<0.0001**

H0: The variable or the interaction of the corresponding column has no significant effect on the dependent variables.

Ha: The variable or the interaction of the corresponding column has a significant effect on the dependent variables.

Position of the substituent: As the computed p-value is lower than the significance level alpha=0.05, one should reject the null hypothesis H0, and accept the alternative hypothesis Ha.

The risk to reject the null hypothesis H0 while it is true is lower than 0.07%.

Presence of the substituent: As the computed p-value is lower than the significance level alpha=0.05, one should reject the null hypothesis H0, and accept the alternative hypothesis Ha.

The risk to reject the null hypothesis H0 while it is true is lower than 0.01%.

Table S4g. Position of the substituent

Variable	Categories
orto position	no
	yes
meta position	no
	yes
para position	no
	yes

3 vs 16 vs 17 vs 18 vs 19	*orto* position	*meta* position	*para* position	*orto* position**meta* position
Lambda	0.949	1.000	0.691	0.000
F Observed values	2.192	0.005	18.299	0.000
DF1	1	1	1	0
DF2	41	41	41	0
F Critical value	4.079	4.079	4.079	0.000
p-value	0.146	0.944	**0.000**	**<0.0001**

H0: The variable or the interaction of the corresponding column has no significant effect on the dependent variables.

Ha: The variable or the interaction of the corresponding column has a significant effect on the dependent variables.

orto position: As the computed p-value is greater than the significance level alpha=0.05, one cannot reject the null hypothesis H0.

The risk to reject the null hypothesis H0 while it is true is 14.64%.

meta position: As the computed p-value is greater than the significance level alpha=0.05, one cannot reject the null hypothesis H0.

The risk to reject the null hypothesis H0 while it is true is 94.37%.

para position: As the computed p-value is lower than the significance level alpha=0.05, one should reject the null hypothesis H0, and accept the alternative hypothesis Ha.

The risk to reject the null hypothesis H0 while it is true is lower than 0.01%.

Table S4h. Type of substituent phenyl ring*presence of substituent - para position

Variable	Categories
Type of substituent	4-CH3
	4-CHO
	4-COOH
	4-Cl
	4-NH3+
	4-NO2
	4-OCH3
	4-OH
	4-OSO3H
	H
Presence of substituent	no
	yes

**ZA vs 3 vs 13 vs 20 vs 22 vs 27 vs 28 vs 29 vs 30 vs 31**	Type of substituent	Presence of substituent	Type of substituen*Presence of substituent
Lambda	0.505	0.000	0.000
F Observed values	12.958	0.000	0.000
DF1	9	0	0
DF2	119	0	0
F Critical value	1.959	0.000	0.000
p-value	**<0.0001**	**<0.0001**	**<0.0001**

H0: The variable or the interaction of the corresponding column has no significant effect on the dependent variables.

Ha: The variable or the interaction of the corresponding column has a significant effect on the dependent variables.

R: As the computed p-value is lower than the significance level alpha=0.05, one should reject the null hypothesis H0, and accept the alternative hypothesis Ha.

The risk to reject the null hypothesis H0 while it is true is lower than 0.01%.

Presence of substituent: As the computed p-value is lower than the significance level alpha=0.05, one should reject the null hypothesis H0, and accept the alternative hypothesis Ha.

The risk to reject the null hypothesis H0 while it is true is lower than 0.01%.

Table S5. Student's t-test analysis performed to compared adhesion data of some relevant ZA-analogues. Test interpretation: H0: The difference between the means is equal to 0. Ha: The difference between the means is different from 0. When the computed p-value is lower than the significance level alpha=0,05, one should reject the null hypothesis H0, and accept the alternative hypothesis Ha.

Table S5a

**3 vs 5**	
Difference	-292376.617
t (Observed value)	-2.362
|t| (Critical value)	2.030
DF	35
p-value (Two-tailed)	**0.024**
alpha	0.05

Table S5b

**13 vs 15**	
Difference	-292376.617
t (Observed value)	-2.362
|t| (Critical value)	2.030
DF	35
p-value (Two-tailed)	**0.024**
alpha	0.05

Table S5c

**ZA vs 2**	
Difference	-549235.802
t (Observed value)	-6.000
|t| (Critical value)	2.028
DF	36
p-value (Two-tailed)	**<0.0001**
alpha	0.05

Table S5d

**3 vs 4**	
Difference	-433775.538
t (Observed value)	-3.270
|t| (Critical value)	2.052
DF	27
p-value (Two-tailed)	**0.003**
alpha	0.05

Table S5e

**13 vs 14**	
Difference	-525131.835
t (Observed value)	-4.322
|t| (Critical value)	2.306
DF	8
p-value (Two-tailed)	**0.003**
alpha	0.05

Table S5f

**3 vs 10**	
Difference	-336851.149
t (Observed value)	-2.596
|t| (Critical value)	2.306
DF	8
p-value (Two-tailed)	**0.032**
alpha	0.05

Table S5g

**3 vs 12**	
Difference	-558792.930
t (Observed value)	-2.862
|t| (Critical value)	2.365
DF	7
p-value (Two-tailed)	**0.024**
alpha	0.05

Table S5h

**3 vs 7**	
Difference	-217515.076
t (Observed value)	-2.060
|t| (Critical value)	2.365
DF	7
p-value (Two-tailed)	**0.078**
alpha	0.1

Table S5i

**13 vs 16**	
Difference	-328125.800
t (Observed value)	-2.818
|t| (Critical value)	2.571
DF	5
p-value (Two-tailed)	**0.037**
alpha	0.05

Table S5j

**6 vs 13**	
Difference	-30080.001
t (Observed value)	0.3701
|t| (Critical value)	0.447
DF	5
p-value (Two-tailed)	0.726
alpha	0.05

Table S5k

**3 vs 18**	
Difference	-200629.444
t (Observed value)	-2.252
|t| (Critical value)	2.306
DF	8
p-value (Two-tailed)	**0.050**
alpha	0.05

Table S5l

**7 vs 9**	
Difference	-261978.646
t (Observed value)	-2.727
|t| (Critical value)	2.306
DF	8
p-value (Two-tailed)	**0.026**
alpha	0.05

Table S5m

**3 vs 17**	
Difference	-525634.932
t (Observed value)	-4.246
|t| (Critical value)	2.306
DF	8
p-value (Two-tailed)	**0.003**
alpha	0.05

Table S5n

**3 vs 23**	
Difference	-490768.913
t (Observed value)	-5.329
|t| (Critical value)	2.306
DF	8
p-value (Two-tailed)	**0.001**
alpha	0.05

Table S5o

3 vs 13	
Difference	-465552.792
t (Observed value)	-9.648
|t| (Critical value)	2.365
DF	7
p-value (Two-tailed)	**<0.0001**
alpha	0.05

Table S5p

3 vs 20	
Difference	-1681915.788
t (Observed value)	-38.820
|t| (Critical value)	2.262
DF	9
p-value (Two-tailed)	**<0.0001**
alpha	0.05

Table S5q

3 vs 22	
Difference	543299.099
t (Observed value)	4.168
|t| (Critical value)	2.306
DF	8
p-value (Two-tailed)	**0.003**
alpha	0.05

Table S5r

3 vs 27	
Difference	-1114932.447
t (Observed value)	-7.993
|t| (Critical value)	2.032
DF	34
p-value (Two-tailed)	**<0.0001**
alpha	0.05

Table S5s

3 vs 28	
Difference	-1094708.04
t (Observed value)	-6.464
|t| (Critical value)	2.028
DF	36
p-value (Two-tailed)	**<0.0001**
alpha	0.05

Table S5t

**3 vs 29**	
Difference	-1006376.93
t (Observed value)	-8.536
|t| (Critical value)	2.045
DF	29
p-value (Two-tailed)	**<0.0001**
alpha	0.05

Table S5u

**3 vs 30**	
Difference	-906811.360
t (Observed value)	-13.430
|t| (Critical value)	2.262
DF	9
p-value (Two-tailed)	**<0.0001**
alpha	0.05

Table S5v

**3 vs 31**	
Difference	-777239.829
t (Observed value)	-23.280
|t| (Critical value)	2.365
DF	7
p-value (Two-tailed)	**<0.0001**
alpha	0.05

Figure S1. Ramachandran plot of the *C. albicans* NAD(P)H quinone oxidoreductase homology models. The three residues in the disallowed region (white area of the plot) do not belong to enzyme portion involved in the interaction with the substrate and FMN.

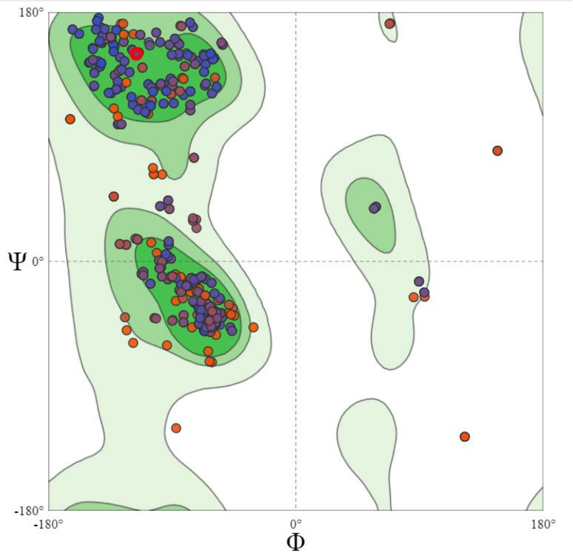

Supplement: Supplementary file 1 [file ao5c03581_si_001.pdf]
